# Some Interval-Valued Intuitionistic Fuzzy Dombi Heronian Mean Operators and their Application for Evaluating the Ecological Value of Forest Ecological Tourism Demonstration Areas

**DOI:** 10.3390/ijerph17030829

**Published:** 2020-01-29

**Authors:** Liangping Wu, Guiwu Wei, Jiang Wu, Cun Wei

**Affiliations:** 1 School of Business, Sichuan Normal University, Chengdu 610101, China; wuliangping6@sicnu.edu.cn; 2 School of Statistics, Southwestern University of Finance and Economics, Chengdu 611130, China; wujiang@swufe.edu.cn (J.W.); weicun1990@163.com (C.W.)

**Keywords:** multiple attribute decision making (MADM), interval-valued intuitionistic fuzzy numbers (IVIFNs), Hamy mean operator, Dombi operation, ecological value, forest ecological tourism demonstration area

## Abstract

With China’s sustained economic development and constant increase in national income, Chinese nationals’ tourism consumption rate increases. As a major Chinese economic development engine, the domestic tourism industry has entered a transition period operation pattern featured by diversified products. Among them, as a new hot spot of the tourism industry in China, ecological tourism has enjoyed rapid development, with great potential. Thus, the ecological value evaluation of forest ecological tourism demonstration areas is very important to the domestic tourism industry. In this paper, we propose some Dombi Heronian mean operators with interval-valued intuitionistic fuzzy numbers (IVIFNs). Then, two MADM (multiple attribute decision making) methods are proposed based on IVIFWDHM (interval-valued intuitionistic fuzzy weighted Dombi Heronian mean) and IVIFWDGHM (interval-valued intuitionistic weighted Dombi geometric Heronian mean) operators. Finally, we gave an experimental case for evaluating the ecological value of forest ecological tourism demonstration area to show the proposed decision methods.

## 1. Introduction

The basic concept of intuitionistic fuzzy sets (IFSs) [1,2] is a useful and effective tool to depict uncertainty and imprecision. Xu [3] proposed some novel correlation coefficients of IFSs. Xu and Yager [4] developed the geometric operators with intuitionistic fuzzy numbers (IFNs). Xu [5] defined some new similarity measures of IFSs for fuzzy MADM (multiple attribute decision making). Li, Gao and Wei [6] defined the Hamy mean (HM) operator and the Dombi Hamy mean (DHM) operator [7,8,9,10] with IFNs. Xu [11] gave the comparison between two IFNs and developed some arithmetic operators for IFNs. Atanassov and Gargov [12] designed the interval-valued IFSs (IVIFSs). Xu and Chen [13] defined the geometric operators with interval-valued intuitionistic fuzzy numbers (IVIFNs). Wu et al. [14] proposed some DHM operators with IVIFNs. Yu et al. [15] extended the prioritized average [16,17,18,19] to develop some novel operators with IVIFNs. Chen [20] presented the likelihood-based functions for solving MADM with IVIFNs. Wei [21] proposed two induced operators with IFNs and IVIFNs. Liu and Teng [22] proposed the normal IVIFNs. Dugenci [23] introduced a novel generalized distance measure for IVIFNs for MAGDM (multiple attribute group decision making). Nguyen [24] discussed some new entropy measures for IVIFSs. Sudharsan and Ezhilmaran [25] presented the weighted arithmetic average operator for investment decision making with IVIFNs. Dammak et al. [26] proposed MADM methods by using elimination et choice transiting reality (ELECTRE) methods [27], IVIFs and possibility theory. Garg et al. [28] presented some novel operators by considering hesitancy degree with IVIFNs. Liu and Li [29] proposed some new power BM operators [30,31,32,33] for MAGDM with IVIFNs. Wang [34] developed Choquet integral operators for fusing the IVIFNs based on Archimedean t-norm. Garg and Arora [35] presented the nonlinear programming TOPSIS (Technique for Order of Preference by Similarity to Ideal Solution) method for MADM. Hashemi et al. [36] proposed the compromise ratio MAGDM model with IVIFNs. Kim et al. [37] proposed the method for evaluating the students’ knowledge obtained in the university e-learning courses with IVIFNs. Liu et al. [38] defined the power MSM (Maclaurin symmetric mean) operator and the weighted power MSM operator with IVIFNs based on the traditional MSM operators [39,40,41]. Garg [42] developed a novel generalized improved score function with IVIFNs. Xia [43] developed the games methods on the basis of Archimedean t-conorm and t-norm with IVIFNs. Chen [44] proposed the IVIF-PROMETHEE (Preference Ranking Organization Method for Enrichment Evaluation) method to cope with MADM. Chen and Han [45] proposed a novel MADM method by applying the nonlinear programming (NLP) model and particle swarm optimization (PSO) methods by using IVIFNs. Liu et al. [46] defined the principal component analysis (PCA) method for IVIFNs. Wei [47], and Chen [48] defined the LINMAP (Linear Programming Technique for Multidimensional Analysis of Preference) method for MADM with IVIFNs. Recently, more and more decision theories with IFNs and IVIFNs are extended to picture fuzzy set [49,50,51,52], Pythagorean fuzzy sets [53,54,55] and other uncertain environments [56,57,58,59,60].

Although, IFSs and IVIFSs have been effectively utilized in some domains, however, all these existing methods are unsuitable to solve the interrelationships among the IVIFNs designed with a variable vector. And Heronian mean (HM) operator and dual Heronian mean (DHM) operator [10] are useful operators which can depict interrelationships with any number of arguments designed by using a variable vector. Therefore, the HM and DHM operators can give some very flexible and robust modes to fuse information in MADM. Thus, we propose some HM operators to overcome these limits. How to aggregate these IVIFNs based the traditional HM operators based on the Dombi operations [61,62,63] is an interesting issue. So, the purpose of our paper is to design some HM operators to solve the MADM with IVIFNs. In order to do so, the rest of our paper is organized as follows. In Section 2, we recall some basic concept of IVIFNs. In Section 3, we propose some HM fused operators with IVIFNs based on Dombi operations. In Section 4, we use an example for evaluating the ecological value of a forest ecological tourism demonstration area with IVIFNs. Section 5 finishes this paper with some conclusions.

## 2. Preliminaries

### 2.1. IFSs and IVIFSs

The concept of IFSs and IVIFSs are introduced.

**Definition** **1** [1,2]**.** *An IFS* F *in* Y *is designed by:*

(1) $$F=\left\{\langle y,\alpha_{F}\left(y\right),\beta_{F}\left(y\right)\rangle |y\in Y\right\}$$

where $\alpha_{F}:\;Y\to \left[0,1\right]$ *and* $\beta_{F}:\;Y\to \left[0,1\right]$, *and* $0\leq \alpha_{F}\left(y\right)+\beta_{F}\left(y\right)\leq 1$, ∀ y∈Y*. The numbers* $\alpha_{F}\left(y\right)$ and $\beta_{F}\left(y\right)$ *represent the membership degree and non- membership degree, respectively, of the element* y *to the set* F*.*

**Definition** **2** [12]**.** *Let* Y *be a universe of discourse, an IVIFS* $\tilde{F}$ *over* Y *is an object defined as follows:*

(2) $$\tilde{F}=\left\{\langle y,{\tilde{\alpha }}_{\tilde{F}}\left(y\right),{\tilde{\beta }}_{\tilde{F}}\left(y\right)\rangle |y\in Y\right\}$$

*where* ${\tilde{\alpha }}_{\tilde{F}}\left(y\right)\subseteq \left[0,1\right]$ *and* ${\tilde{\beta }}_{\tilde{F}}\left(y\right)\subseteq \left[0,1\right]$ *are interval numbers, and* $0\leq \sup\left({\tilde{\alpha }}_{\tilde{F}}\left(y\right)\right)+\sup\left({\tilde{\beta }}_{\tilde{F}}\left(y\right)\right)\leq 1$*,* ∀ y∈Y*. For convenience, let* ${\tilde{\alpha }}_{\tilde{F}}\left(y\right)=\left[b,d\right]$*,* ${\tilde{\beta }}_{\tilde{F}}\left(y\right)=[e,g]$*, so* $\tilde{\delta }=\left(\left[b,d\right],\left[e,g\right]\right)$ *is an IVIFN.*

**Definition** **3** [64]**.** *Let* $\tilde{\delta }=\left(\left[b,d\right],\left[e,g\right]\right)$ *be an IVIFN, a score function* S *is defined:*

(3) $$S\left(\tilde{\delta }\right)=\frac{b-e+d-g}{2},S\left(\tilde{\delta }\right)\in \left[-1,1\right].$$

**Definition** **4** [64]**.** *Let* $\tilde{\delta }=\left(\left[b,d\right],\left[e,g\right]\right)$ *be an IVIFN, an accuracy function* H *can be defined:*

(4) $$H\left(\tilde{\delta }\right)=\frac{b+e+d+g}{2},H\left(\tilde{\delta }\right)\in \left[0,1\right]$$

*to evaluate the degree of accuracy of the IVIFN* $\tilde{\delta }=\left(\left[b,d\right],\left[e,g\right]\right)$.

**Definition** **5** [64]**.** *Let* ${\tilde{\delta }}_{1}=\left(\left[b_{1},d_{1}\right],\left[e_{1},g_{1}\right]\right)$ *and* ${\tilde{\delta }}_{2}=\left(\left[b_{2},d_{2}\right],\left[e_{2},g_{2}\right]\right)$ *be two IVIFNs,* $S\left({\tilde{\delta }}_{1}\right)=\frac{b_{1}-e_{1}+d_{1}-g_{1}}{2}$ *and* $S\left({\tilde{\delta }}_{2}\right)=\frac{b_{2}-e_{2}+d_{2}-g_{2}}{2}$ *be the scores of* ${\tilde{\delta }}_{1}$ and ${\tilde{\delta }}_{2}$*, respectively, and let* $H\left({\tilde{\delta }}_{1}\right)=\frac{b_{1}+e_{1}+d_{1}+g_{1}}{2}$ *and* $H\left({\tilde{\delta }}_{2}\right)=\frac{b_{2}+e_{2}+d_{2}+g_{2}}{2}$ *be the accuracy degrees of* ${\tilde{\delta }}_{1}$ *and* ${\tilde{\delta }}_{2}$*, respectively, then if* $S\left({\tilde{\delta }}_{1}\right)<S\left({\tilde{\delta }}_{2}\right)$*, then* ${\tilde{\delta }}_{1}<{\tilde{\delta }}_{2}$*; if* $S\left({\tilde{\delta }}_{1}\right)=S\left({\tilde{\delta }}_{2}\right)$*, then (1) if* $H\left({\tilde{\delta }}_{1}\right)=H\left({\tilde{\delta }}_{2}\right)$*, then* ${\tilde{\delta }}_{1}={\tilde{\delta }}_{2}$*; (2) if* $H\left({\tilde{\delta }}_{1}\right)<H\left({\tilde{\delta }}_{2}\right)$*, then* ${\tilde{\delta }}_{1}<{\tilde{\delta }}_{2}$

**Definition** **6** [64]**.** *For two IVIFNs* ${\tilde{\delta }}_{1}=\left(\left[b_{1},d_{1}\right],\left[e_{1},g_{1}\right]\right)$ *and* ${\tilde{\delta }}_{2}=\left(\left[b_{2},d_{2}\right],\left[e_{2},g_{2}\right]\right)$*, the operational laws are defined:*

*(1)*  ${\tilde{\delta }}_{1}⊕{\tilde{\delta }}_{2}=\left(\left[b_{1}+b_{2}-b_{1}b_{2},d_{1}+d_{2}-d_{1}d_{2}\right],\left[e_{1}e_{2},g_{1}g_{2}\right]\right)$;
*(2)*  ${\tilde{\delta }}_{1}⊗{\tilde{\delta }}_{2}=\left(\left[b_{1}b_{2},d_{1}d_{2}\right],\left[e_{1}+e_{2}-e_{1}e_{2},g_{1}+g_{2}-g_{1}g_{2}\right]\right)$;
*(3)*  $\lambda {\tilde{\delta }}_{1}=\left(\left[1-{\left(1-b_{1}\right)}^{\lambda },1-{\left(1-d_{1}\right)}^{\lambda }\right],\left[e_{1}^{\lambda },g_{1}^{\lambda }\right]\right),\lambda >0$;
*(4)*  ${\left({\tilde{\delta }}_{1}\right)}^{\lambda }=\left(\left[b_{1}^{\lambda },d_{1}^{\lambda }\right],\left[1-{\left(1-e_{1}\right)}^{\lambda },1-{\left(1-g_{1}\right)}^{\lambda }\right]\right),\lambda >0$.

### 2.2. HM Operator

Hara, Uchiyama and Takahasi [65] proposed the Heronian mean (HM) operator.

**Definition** **7** [65]**.** *The Heronian mean (HM) operator is defined:*

(5) $${\mathrm{HM}}^{p,q}\left(\delta_{1},\delta_{2},\cdots ,\delta_{n}\right)={\left(\frac{2}{n(n+1)}\sum_{i=1}^{n}\sum_{j=i}^{n}\delta_{i}^{p}\delta_{j}^{q}\right)}^{\frac{1}{p+q}}$$

*where p, q ≥ 0, then* $\delta_{i}\left(i=1,2,\cdots ,n\right)$ *be a series of crisp numbers.*

### 2.3. Dombi Operations of IVIFNs

**Definition** **8** [61]**.** *Dombi [61] proposed the Dombi T-norm and T-conorm:*

(6) $$D\left(t,s\right)=\frac{1}{1+{\left({\left(\frac{1-t}{t}\right)}^{\gamma }+{\left(\frac{1-s}{s}\right)}^{\gamma }\right)}^{1/\gamma }}$$

(7) $$D^{c}\left(t,s\right)=1-\frac{1}{1+{\left({\left(\frac{t}{1-t}\right)}^{\gamma }+{\left(\frac{s}{1-s}\right)}^{\gamma }\right)}^{1/\gamma }}$$

*where* γ>0, (t,s)∈[0,1].

Based on the Dombi T-norm and T-conorm, we can give the operational rules of IVIFNs.

**Definition** **9.** *For two IVIFNs*${\tilde{\delta }}_{1}=\left(\left[b_{1},d_{1}\right],\left[e_{1},g_{1}\right]\right)$*and*${\tilde{\delta }}_{2}=\left(\left[b_{2},d_{2}\right],\left[e_{2},g_{2}\right]\right)$*,*γ>0, *the Dombi operational laws are defined:*

*(1)*  ${\tilde{\delta }}_{1}⊕{\tilde{\delta }}_{2}=\left(\begin{array}{l} \left[1-\frac{1}{1+{\left({\left(\frac{b_{1}}{1-b_{1}}\right)}^{\gamma }+{\left(\frac{b_{2}}{1-b_{2}}\right)}^{\gamma }\right)}^{\frac{1}{\gamma }}},1-\frac{1}{1+{\left({\left(\frac{d_{1}}{1-d_{1}}\right)}^{\gamma }+{\left(\frac{d_{2}}{1-d_{2}}\right)}^{\gamma }\right)}^{\frac{1}{\gamma }}}\right],\\ \left[\frac{1}{1+{\left({\left(\frac{1-e_{1}}{e_{1}}\right)}^{\gamma }+{\left(\frac{1-e_{2}}{e_{2}}\right)}^{\gamma }\right)}^{\frac{1}{\gamma }}},\frac{1}{1+{\left({\left(\frac{1-g_{1}}{g_{1}}\right)}^{\gamma }+{\left(\frac{1-g_{2}}{g_{2}}\right)}^{\gamma }\right)}^{\frac{1}{\gamma }}}\right] \end{array}\right);$
*(2)*  ${\tilde{\delta }}_{1}⊗{\tilde{\delta }}_{2}=\left(\begin{array}{l} \left[\frac{1}{1+{\left({\left(\frac{1-b_{1}}{b_{1}}\right)}^{\gamma }+{\left(\frac{1-b_{2}}{b_{2}}\right)}^{\gamma }\right)}^{\frac{1}{\gamma }}},\frac{1}{1+{\left({\left(\frac{1-d_{1}}{d_{1}}\right)}^{\gamma }+{\left(\frac{1-d_{2}}{d_{2}}\right)}^{\gamma }\right)}^{\frac{1}{\gamma }}}\right],\\ \left[1-\frac{1}{1+{\left({\left(\frac{e_{1}}{1-e_{1}}\right)}^{\gamma }+{\left(\frac{e_{2}}{1-e_{2}}\right)}^{\gamma }\right)}^{\frac{1}{\gamma }}},1-\frac{1}{1+{\left({\left(\frac{g_{1}}{1-g_{1}}\right)}^{\gamma }+{\left(\frac{g_{2}}{1-g_{2}}\right)}^{\gamma }\right)}^{\frac{1}{\gamma }}}\right] \end{array}\right);$
*(3)*  $n{\tilde{\delta }}_{1}=\left(\left[1-\frac{1}{1+{\left(n{\left(\frac{b_{1}}{1-b_{1}}\right)}^{\gamma }\right)}^{\frac{1}{\gamma }}},1-\frac{1}{1+{\left(n{\left(\frac{d_{1}}{1-d_{1}}\right)}^{\gamma }\right)}^{\frac{1}{\gamma }}}\right],\left[\frac{1}{1+{\left(n{\left(\frac{1-e_{1}}{e_{1}}\right)}^{\gamma }\right)}^{\frac{1}{\gamma }}},\frac{1}{1+{\left(n{\left(\frac{1-g_{1}}{g_{1}}\right)}^{\gamma }\right)}^{\frac{1}{\gamma }}}\right]\right);$
*(4)*  ${\left({\tilde{\delta }}_{1}\right)}^{n}=\left(\left[\frac{1}{1+{\left(n{\left(\frac{1-b_{1}}{b_{1}}\right)}^{\gamma }\right)}^{\frac{1}{\gamma }}},\frac{1}{1+{\left(n{\left(\frac{1-d_{1}}{d_{1}}\right)}^{\gamma }\right)}^{\frac{1}{\gamma }}}\right],\left[1-\frac{1}{1+{\left(n{\left(\frac{e_{1}}{1-e_{1}}\right)}^{\gamma }\right)}^{\frac{1}{\gamma }}},1-\frac{1}{1+{\left(n{\left(\frac{g_{1}}{1-g_{1}}\right)}^{\gamma }\right)}^{\frac{1}{\gamma }}}\right]\right).$

## 3. Some Dombi Heronian Mean Operators with IVIFNs

### 3.1. The IVIFDHM Operator

Based on the HM operator and Dombi operation rules, the IVIFDHM operator is defined:

**Definition** **10.** *Let* ${\tilde{\delta }}_{i}=\left(\left[b_{i},d_{i}\right],\left[e_{i},g_{i}\right]\right)\left(i=1,2,\ldots ,n\right)$ *be a set of IVIFNs. The IVIFDHM operator is:*

(8) $${\mathrm{IVIFDHM}}^{p,q}({\tilde{\delta }}_{1},{\tilde{\delta }}_{2},\ldots ,{\tilde{\delta }}_{n})={\left(\frac{2}{n(n+1)}\overset{n}{\underset{i=1}{⊕}}\overset{n}{\underset{j=i}{⊕}}\left({\tilde{\delta }}_{i}^{p}⊗{\tilde{\delta }}_{j}^{q}\right)\right)}^{\frac{1}{p+q}}$$

**Theorem** **1.** *Let*${\tilde{\delta }}_{i}=\left(\left[b_{i},d_{i}\right],\left[e_{i},g_{i}\right]\right)\left(i=1,2,\ldots ,n\right)$*be a set of IVIFNs and*p,q≥0,γ>0. *The fused value by IVIFDHM operators is also an IVIFN, and:*

${\mathrm{IVIFDHM}}^{p,q}({\tilde{\delta }}_{1},{\tilde{\delta }}_{2},\ldots ,{\tilde{\delta }}_{n})={\left(\frac{2}{n(n+1)}\overset{n}{\underset{i=1}{⊕}}\overset{n}{\underset{j=i}{⊕}}\left({\tilde{\delta }}_{i}^{p}⊗{\tilde{\delta }}_{j}^{q}\right)\right)}^{\frac{1}{p+q}}$

(9) =([1(1+(n(n+1)2(p+q)×(1∑i=1n∑j=in1pBiγ+qBjγ))1γ),1(1+(n(n+1)2(p+q)×(1∑i=1n∑j=in1pDiγ+qDjγ))1γ)],[1−1(1+(n(n+1)2(p+q)×(1∑i=1n∑j=in1pEiγ+qEjγ))1γ),1−1(1+(n(n+1)2(p+q)×(1∑i=1n∑j=in1pGiγ+qGjγ))1γ)])

*where* $B_{i}=\frac{1-b_{i}}{b_{i}},D_{i}=\frac{1-d_{i}}{d_{i}},E_{i}=\frac{e_{i}}{1-e_{i}},G_{i}=\frac{g_{i}}{1-g_{i}},B_{j}=\frac{1-b_{j}}{b_{j}},D_{j}=\frac{1-d_{j}}{d_{j}},E_{j}=\frac{e_{j}}{1-e_{j}},G_{j}=\frac{g_{j}}{1-g_{j}}$

**Proofs.**   

(10) $$\begin{array}{l} {\tilde{\delta }}_{i}^{p}=\left(\left[\frac{1}{1+{\left(p{\left(\frac{1-b_{i}}{b_{i}}\right)}^{\gamma }\right)}^{\frac{1}{\gamma }}},\frac{1}{1+{\left(p{\left(\frac{1-d_{i}}{d_{i}}\right)}^{\gamma }\right)}^{\frac{1}{\gamma }}}\right],\left[1-\frac{1}{1+{\left(p{\left(\frac{e_{i}}{1-e_{i}}\right)}^{\gamma }\right)}^{\frac{1}{\gamma }}},\frac{1}{1+{\left(p{\left(\frac{g_{i}}{1-g_{i}}\right)}^{\gamma }\right)}^{\frac{1}{\gamma }}}\right]\right),\\ {\tilde{\delta }}_{j}^{q}=\left(\left[\frac{1}{1+{\left(q{\left(\frac{1-b_{j}}{b_{j}}\right)}^{\gamma }\right)}^{\frac{1}{\gamma }}},\frac{1}{1+{\left(q{\left(\frac{1-d_{j}}{d_{j}}\right)}^{\gamma }\right)}^{\frac{1}{\gamma }}}\right],\left[1-\frac{1}{1+{\left(q{\left(\frac{e_{j}}{1-e_{j}}\right)}^{\gamma }\right)}^{\frac{1}{\gamma }}},\frac{1}{1+{\left(q{\left(\frac{g_{j}}{1-g_{j}}\right)}^{\gamma }\right)}^{\frac{1}{\gamma }}}\right]\right) \end{array}$$

Let $B_{i}=\frac{1-b_{i}}{b_{i}},D_{i}=\frac{1-d_{i}}{d_{i}},E_{i}=\frac{e_{i}}{1-e_{i}},G_{i}=\frac{g_{i}}{1-g_{i}},B_{j}=\frac{1-b_{j}}{b_{j}},D_{j}=\frac{1-d_{j}}{d_{j}},E_{j}=\frac{e_{j}}{1-e_{j}},G_{j}=\frac{g_{j}}{1-g_{j}}$,

Then,

(11) $$\begin{array}{l} {\tilde{\delta }}_{i}^{p}=\left(\left[\frac{1}{1+{\left(pB_{i}^{\gamma }\right)}^{\frac{1}{\gamma }}},\frac{1}{1+{\left(pD_{i}^{\gamma }\right)}^{\frac{1}{\gamma }}}\right],\left[1-\frac{1}{1+{\left(pE_{i}^{\gamma }\right)}^{\frac{1}{\gamma }}},1-\frac{1}{1+{\left(pG_{i}^{\gamma }\right)}^{\frac{1}{\gamma }}}\right]\right),\\ {\tilde{\delta }}_{j}^{q}=\left(\left[\frac{1}{1+{\left(qB_{j}^{\gamma }\right)}^{\frac{1}{\gamma }}},\frac{1}{1+{\left(qD_{j}^{\gamma }\right)}^{\frac{1}{\gamma }}}\right],\left[1-\frac{1}{1+{\left(qE_{j}^{\gamma }\right)}^{\frac{1}{\gamma }}},1-\frac{1}{1+{\left(qG_{j}^{\gamma }\right)}^{\frac{1}{\gamma }}}\right]\right) \end{array}$$

Thus,

(12) $${\tilde{\delta }}_{i}^{p}⊗{\tilde{\delta }}_{j}^{q}=\left(\begin{array}{l} \left[\frac{1}{1+{\left(pB_{i}^{\gamma }+qB_{j}^{\gamma }\right)}^{\frac{1}{\gamma }}},\frac{1}{1+{\left(pD_{i}^{\gamma }+qD_{j}^{\gamma }\right)}^{\frac{1}{\gamma }}}\right],\\ \left[1-\frac{1}{1+{\left(pE_{i}^{\gamma }+qE_{j}^{\gamma }\right)}^{\frac{1}{\gamma }}},1-\frac{1}{1+{\left(pG_{i}^{\gamma }+qG_{j}^{\gamma }\right)}^{\frac{1}{\gamma }}}\right] \end{array}\right)$$

Thereafter,

(13) $$\overset{n}{\underset{j=i}{⊕}}\left({\tilde{\delta }}_{i}^{p}⊗{\tilde{\delta }}_{j}^{q}\right)=\left(\begin{array}{l} \left[1-\frac{1}{\left(1+{\left(\sum_{j=i}^{n}\frac{1}{pB_{i}^{\gamma }+qB_{j}^{\gamma }}\right)}^{\frac{1}{\gamma }}\right)},1-\frac{1}{\left(1+{\left(\sum_{j=i}^{n}\frac{1}{pD_{i}^{\gamma }+qD_{j}^{\gamma }}\right)}^{\frac{1}{\gamma }}\right)}\right],\\ \left[\frac{1}{\left(1+{\left(\sum_{j=i}^{n}\frac{1}{pE_{i}^{\gamma }+qE_{j}^{\gamma }}\right)}^{\frac{1}{\gamma }}\right)},\frac{1}{\left(1+{\left(\sum_{j=i}^{n}\frac{1}{pG_{i}^{\gamma }+qG_{j}^{\gamma }}\right)}^{\frac{1}{\gamma }}\right)}\right] \end{array}\right)$$

And,

(14) $$\begin{array}{l} \overset{n}{\underset{i=1}{⊕}}\overset{n}{\underset{j=i}{⊕}}\left({\tilde{\delta }}_{i}^{p}⊗{\tilde{\delta }}_{j}^{q}\right)\\ =\left(\begin{array}{l} \left[1-\frac{1}{\left(1+{\left(\sum_{i=1}^{n}\sum_{j=i}^{n}\frac{1}{pB_{i}^{\gamma }+qB_{j}^{\gamma }}\right)}^{\frac{1}{\gamma }}\right)},1-\frac{1}{\left(1+{\left(\sum_{i=1}^{n}\sum_{j=i}^{n}\frac{1}{pD_{i}^{\gamma }+qD_{j}^{\gamma }}\right)}^{\frac{1}{\gamma }}\right)}\right],\\ \left[\frac{1}{\left(1+{\left(\sum_{i=1}^{n}\sum_{j=i}^{n}\frac{1}{pE_{i}^{\gamma }+qE_{j}^{\gamma }}\right)}^{\frac{1}{\gamma }}\right)},\frac{1}{\left(1+{\left(\sum_{i=1}^{n}\sum_{j=i}^{n}\frac{1}{pG_{i}^{\gamma }+qG_{j}^{\gamma }}\right)}^{\frac{1}{\gamma }}\right)}\right] \end{array}\right) \end{array}$$

Therefore,

(15) $$\begin{array}{l} \frac{2}{n(n+1)}\overset{n}{\underset{i=1}{⊕}}\overset{n}{\underset{j=i}{⊕}}\left({\tilde{\delta }}_{i}^{p}⊗{\tilde{\delta }}_{j}^{q}\right)\\ =\left(\begin{array}{l} \left[1-\frac{1}{\left(1+{\left(\frac{2}{n(n+1)}\sum_{i=1}^{n}\sum_{j=i}^{n}\frac{1}{pB_{i}^{\gamma }+qB_{j}^{\gamma }}\right)}^{\frac{1}{\gamma }}\right)},1-\frac{1}{\left(1+{\left(\frac{2}{n(n+1)}\sum_{i=1}^{n}\sum_{j=i}^{n}\frac{1}{pD_{i}^{\gamma }+qD_{j}^{\gamma }}\right)}^{\frac{1}{\gamma }}\right)}\right],\\ \left[\frac{1}{\left(1+{\left(\frac{2}{n(n+1)}\sum_{i=1}^{n}\sum_{j=i}^{n}\frac{1}{pE_{i}^{\gamma }+qE_{j}^{\gamma }}\right)}^{\frac{1}{\gamma }}\right)},\frac{1}{\left(1+{\left(\frac{2}{n(n+1)}\sum_{i=1}^{n}\sum_{j=i}^{n}\frac{1}{pG_{i}^{\gamma }+qG_{j}^{\gamma }}\right)}^{\frac{1}{\gamma }}\right)}\right] \end{array}\right) \end{array}$$

Furthermore,

(16) (2n(n+1)⊕i=1n⊕j=in(δ˜ip⊗δ˜jq))1p+q=([1(1+(n(n+1)2(p+q)×(1∑i=1n∑j=in1pBiγ+qBjγ))1γ),1(1+(n(n+1)2(p+q)×(1∑i=1n∑j=in1pDiγ+qDjγ))1γ)],[1−1(1+(n(n+1)2(p+q)×(1∑i=1n∑j=in1pEiγ+qEjγ))1γ),1−1(1+(n(n+1)2(p+q)×(1∑i=1n∑j=in1pGiγ+qGjγ))1γ)])

Thus, (9) is right. □

**Example** **1.** *Let* ${\tilde{\delta }}_{1}=\left(\left[0.2,0.5\right],\left[0.3,0.5\right]\right),{\tilde{\delta }}_{2}=\left(\left[0.3,0.6\right],\left[0.1,0.3\right]\right)$ *, and* ${\tilde{\delta }}_{3}=\left(\left[0.1,0.2\right],\left[0.2,0.4\right]\right)$ *be three IVIFNs, and,* p=2,q=1,γ=3 *. Then, we use the IVIFDHM operator to fuse three IVIFNs.*

First,

(17) $$\begin{array}{l} \sum_{i=1}^{n}\sum_{j=i}^{n}\frac{1}{pB_{i}^{\gamma }+qB_{j}^{\gamma }}=\sum_{i=1}^{3}\sum_{j=i}^{3}\frac{1}{2\times B_{i}^{3}+1\times B_{j}^{3}},\\ =\frac{1}{2\times B_{1}^{3}+1\times B_{1}^{3}}+\frac{1}{2\times B_{1}^{3}+1\times B_{2}^{3}}+\frac{1}{2\times B_{1}^{3}+1\times B_{3}^{3}},\\ +\frac{1}{2\times B_{2}^{3}+1\times B_{2}^{3}}+\frac{1}{2\times B_{2}^{3}+1\times B_{3}^{3}}+\frac{1}{2\times B_{3}^{3}+1\times B_{3}^{3}},\\ =\frac{1}{2\times {\left(\frac{1-0.2}{0.2}\right)}^{3}+1\times {\left(\frac{1-0.2}{0.2}\right)}^{3}}+\frac{1}{2\times {\left(\frac{1-0.2}{0.2}\right)}^{3}+1\times {\left(\frac{1-0.3}{0.3}\right)}^{3}}+\frac{1}{2\times {\left(\frac{1-0.2}{0.2}\right)}^{3}+1\times {\left(\frac{1-0.1}{0.1}\right)}^{3}},\\ +\frac{1}{2\times {\left(\frac{1-0.3}{0.3}\right)}^{3}+1\times {\left(\frac{1-0.3}{0.3}\right)}^{3}}+\frac{1}{2\times {\left(\frac{1-0.3}{0.3}\right)}^{3}+1\times {\left(\frac{1-0.1}{0.1}\right)}^{3}}+\frac{1}{2\times {\left(\frac{1-0.1}{0.1}\right)}^{3}+1\times {\left(\frac{1-0.1}{0.1}\right)}^{3}},\\ =0.0415 \end{array}$$

Then, we have,

(18) 1(1+(n(n+1)2(p+q)×(1∑i=1n∑j=in1pBiγ+qBjγ))1λ)=1(1+(3(3+1)2(2+1)×(1∑i=13∑j=i312×Bi3+1×Bj3))13)=0.2156

Similarly, we have,

(19) 1(1+(n(n+1)2(p+q)×(1∑i=1n∑j=in1pDiγ+qDjγ))1γ)=1(1+(3(3+1)2(2+1)×(1∑i=1n∑j=in12×Di3+1×Dj3))13)=0.4970

And,

(20) 1−1(1+(n(n+1)2(p+q)×(1∑i=1n∑j=in1pEiγ+qEjγ))1γ)=1−1(1+(3(3+1)2(2+1)×(1∑i=1n∑j=in12×Ei3+1×Ej3))13)=0.1535

And,

(21) 1−1(1+(n(n+1)2(p+q)×(1∑i=1n∑j=in1pGiγ+qGjγ))1γ)=1−1(1+(3(3+1)2(2+1)×(1∑i=13∑j=i312×Gi3+1×Gj3))13)=0.3789

Finally, ${\mathrm{IVIFDHM}}^{2,1}({\tilde{\delta }}_{1},{\tilde{\delta }}_{2},{\tilde{\delta }}_{3})=\left(\left[0.2048,0.4743\right],\left[0.1659,0.3875\right]\right)$

Then we list some good properties of IVIFDHM operator.

**Property** **1.** *(Idempotency) If* ${\tilde{\delta }}_{i}=\left(\left[b_{i},d_{i}\right],\left[e_{i},g_{i}\right]\right)\left(i=1,2,\ldots ,n\right)=\tilde{\delta }$ *are equal, then,*

(22) $${\mathrm{IVIFDHM}}^{p,q}({\tilde{\delta }}_{1},{\tilde{\delta }}_{2},\ldots ,{\tilde{\delta }}_{n})=\tilde{\delta }$$

**Proofs.**   

Let ${\tilde{\delta }}_{i}=\left(\left[b_{i},d_{i}\right],\left[e_{i},g_{i}\right]\right)\left(i=1,2,\ldots ,n\right)=\tilde{\delta }=\left(\left[b,d\right],\left[e,g\right]\right)$, so $B=B_{i}=B_{j}=\frac{1-b}{b}$, suppose ${\mathrm{IVIFDHM}}^{p,q}({\tilde{\delta }}_{1},{\tilde{\delta }}_{2},\ldots ,{\tilde{\delta }}_{n})=\left(\left[b_{\alpha },d_{\alpha }\right],\left[e_{\alpha },g_{\alpha }\right]\right)$, we have:

(23) bα=1(1+(n(n+1)2(p+q)×(1∑i=1n∑j=in1pBiγ+qBjγ))1γ)=1(1+(n(n+1)2(p+q)×(1∑i=1n∑j=in1(p+q)Bγ))1γ)=1(1+(n(n+1)2(p+q)×(1n(n+1)2(p+q)Bγ))1γ)=1(1+(Bγ)1γ)=11+B=b

Similarly, we may prove that: $d_{\alpha }=d,e_{\alpha }=e,g_{\alpha }=g$.

So ${\mathrm{IVIFDHM}}^{p,q}({\tilde{\delta }}_{1},{\tilde{\delta }}_{2},\ldots ,{\tilde{\delta }}_{n})=\left(\left[b,d\right],\left[e,g\right]\right)=\tilde{\delta }$. Property 1 is proved. □

**Property** **2.** *(Monotonicity) Let* ${\tilde{\delta }}_{i}=\left(\left[b_{i},d_{i}\right],\left[e_{i},g_{i}\right]\right)(i=1,2,\ldots ,n)$ *, and* ${\tilde{\theta }}_{i}=\left(\left[r_{i},h_{i}\right],\left[m_{i},f_{i}\right]\right)$ (i=1,2,…,n) *be two sets of IVIFNs. If* $b_{i}\leq r_{i},d_{i}\leq h_{i}\;and\;e_{i}\geq m_{i},g_{i}\geq f_{i}$ *hold for all* i *, then,*

(24) $${\mathrm{IVIFDHM}}^{p,q}({\tilde{\delta }}_{1},{\tilde{\delta }}_{2},\ldots ,{\tilde{\delta }}_{n})\leq {\mathrm{IVIFDHM}}^{p,q}({\tilde{\theta }}_{1},{\tilde{\theta }}_{2},\ldots ,{\tilde{\theta }}_{n})$$

**Proofs.**   

Let ${\mathrm{IVIFDHM}}^{p,q}({\tilde{\delta }}_{1},{\tilde{\delta }}_{2},\ldots ,{\tilde{\delta }}_{n})={\tilde{\varphi }}_{\alpha }=\left(\left[b_{\alpha },d_{\alpha }\right],\left[e_{\alpha },g_{\alpha }\right]\right)$, and ${\mathrm{IVIFDHM}}^{p,q}({\tilde{\theta }}_{1},{\tilde{\theta }}_{2},\ldots ,{\tilde{\theta }}_{n})={\tilde{\theta }}_{\alpha }=\left(\left[r_{\alpha },h_{\alpha }\right],\left[m_{\alpha },f_{\alpha }\right]\right)$

Since $b_{i}\leq r_{i},d_{i}\leq h_{i}\;and\;e_{i}\geq m_{i},g_{i}\geq f_{i}$, then we have:

(25) $$B_{i}=\frac{1-b_{i}}{b_{i}}\geq R_{i}=\frac{1-r_{i}}{r_{i}},D_{i}=\frac{1-d_{i}}{d_{i}}\geq H_{i}=\frac{1-h_{i}}{h_{i}},E_{i}=\frac{e_{i}}{1-e_{i}}\geq M_{i}=\frac{m_{i}}{1-m_{i}},G_{i}=\frac{g_{i}}{1-g_{i}}\geq F_{i}=\frac{f_{i}}{1-f_{i}}$$

Therefore,

(26) bα=1(1+(n(n+1)2(p+q)×(1∑i=1n∑j=in1pBiγ+qBjγ))1γ)≤rα=1(1+(n(n+1)2(p+q)×(1∑i=1n∑j=in1pRiγ+qRjγ))1γ)

And,

(27) dα=1−1(1+(n(n+1)2(p+q)×(1∑i=1n∑j=in1pDiγ+qDjγ))1γ)≥hα=1−1(1+(n(n+1)2(p+q)×(1∑i=1n∑j=in1pHiγ+qHjγ))1γ)

Similarly, we have:

(28) $$e_{\alpha }\leq m_{\alpha }\;and\;g_{\alpha }\geq f_{\alpha }$$

So,

(29) $$S\left({\tilde{\delta }}_{\alpha }\right)=\frac{b_{\alpha }-e_{\alpha }+d_{\alpha }-g_{\alpha }}{2}\leq S\left({\tilde{\theta }}_{\alpha }\right)=\frac{r_{\alpha }-m_{\alpha }+h_{\alpha }-f_{\alpha }}{2}$$

Thus, ${\mathrm{IVIFDHM}}^{p,q}({\tilde{\delta }}_{1},{\tilde{\delta }}_{2},\ldots ,{\tilde{\delta }}_{n})={\tilde{\delta }}_{\alpha }\leq {\mathrm{IVIFDHM}}^{p,q}({\tilde{\theta }}_{1},{\tilde{\theta }}_{2},\ldots ,{\tilde{\theta }}_{n})={\tilde{\theta }}_{\alpha }$. Property 2 is proved. □

**Property** **3.** *(Boundedness) Let* ${\tilde{\delta }}_{i}=\left(\left[b_{i},d_{i}\right],\left[e_{i},g_{i}\right]\right)(i=1,2,\ldots ,n)$ *be a set of IVIFNs. If* ${\tilde{\delta }}^{+}=\left(\left(\left[\max_{i}\left(b_{i}\right),\max_{i}\left(d_{i}\right)\right],\left[\min_{i}\left(e_{i}\right),\min_{i}\left(g_{i}\right)\right]\right)\right)$ *and* ${\tilde{\delta }}^{-}=\left(\left[\min_{i}\left(b_{i}\right),\min_{i}\left(d_{i}\right)\right],\left[\max_{i}\left(e_{i}\right),\max_{i}\left(g_{i}\right)\right]\right)$ *, then,*

(30) $${\tilde{\delta }}^{-}\leq {\mathrm{IVIFDWHM}}^{p,q}({\tilde{\delta }}_{1},{\tilde{\delta }}_{2},\ldots ,{\tilde{\delta }}_{n})\leq {\tilde{\delta }}^{+}$$

**Proofs.**   

According to Property 1, we have:

(31) $${\mathrm{IVIFDHM}}^{p,q}({\tilde{\delta }}^{-},{\tilde{\delta }}^{-},\ldots ,{\tilde{\delta }}^{-})={\tilde{\delta }}^{-},{\mathrm{IVIFDHM}}^{p,q}({\tilde{\delta }}^{+},{\tilde{\delta }}^{+},\ldots ,{\tilde{\delta }}^{+})={\tilde{\delta }}^{+}$$

Therefore,

(32) $${\mathrm{IVIFDHM}}^{p,q}({\tilde{\delta }}^{-},{\tilde{\delta }}^{-},\ldots ,{\tilde{\delta }}^{-})\leq {\mathrm{IVIFDHM}}^{p,q}({\tilde{\delta }}_{1},{\tilde{\delta }}_{2},\ldots ,{\tilde{\delta }}_{n})\leq {\mathrm{IVIFDHM}}^{p,q}({\tilde{\delta }}^{+},{\tilde{\delta }}^{+},\ldots ,{\tilde{\delta }}^{+})$$

Then, Property 3 is proved. □

### 3.2. The IVIFWDHM Operator

In real MADM, it’s very important to pay attention to attribute weights. Thus, we must define the interval-valued intuitionistic fuzzy weighted Dombi Heronian mean (IVIFWDHM) operator.

**Definition** **11.** *Let* ${\tilde{\delta }}_{i}=\left(\left[b_{i},d_{i}\right],\left[e_{i},g_{i}\right]\right)\left(i=1,2,\ldots ,n\right)$ *be a set of IVIFNs with weight* $w_{i}={\left(w_{1},w_{2},\ldots ,w_{n}\right)}^{T}$ *, and satisfying* $w_{i}\in \left[0,1\right]$ *and* $\sum_{i=1}^{n}w_{i}=1$ *. Then the IVIFWDHM operator is:*

(33) $${\mathrm{IVIFWDHM}}_{w}^{p,q}({\tilde{\delta }}_{1},{\tilde{\delta }}_{2},\ldots ,{\tilde{\delta }}_{n})={\left(\frac{2}{n(n+1)}\overset{n}{\underset{i=1}{⊕}}\overset{n}{\underset{j=i}{⊕}}\left({\left(w_{i}{\tilde{\delta }}_{i}\right)}^{p}⊗{\left(w_{j}{\tilde{\delta }}_{j}\right)}^{q}\right)\right)}^{\frac{1}{p+q}}$$

**Theorem** **2.** *Let* ${\tilde{\delta }}_{i}=\left(\left[b_{i},d_{i}\right],\left[e_{i},g_{i}\right]\right)\left(i=1,2,\ldots ,n\right)$ *be a set of IVIFNs, and* p,q≥0,γ>0 *. The fused value by IVIFWDHM operators is also an IVIFN, and:*

${\mathrm{IVIFWDHM}}_{w}^{p,q}({\tilde{\delta }}_{1},{\tilde{\delta }}_{2},\ldots ,{\tilde{\delta }}_{n})={\left(\frac{2}{n(n+1)}\overset{n}{\underset{i=1}{⊕}}\overset{n}{\underset{j=i}{⊕}}\left({\left(w_{i}{\tilde{\delta }}_{i}\right)}^{p}⊗{\left(w_{j}{\tilde{\delta }}_{j}\right)}^{q}\right)\right)}^{\frac{1}{p+q}}$

(34) =([1(1+(n(n+1)2(p+q)×1∑i=1n∑j=in(1p/(wiBiγ)+q/(wjBjγ)))1γ),1(1+(n(n+1)2(p+q)×1∑i=1n∑j=in(1p/(wiDiγ)+q/(wjDjγ)))1γ)],[1−1(1+(n(n+1)2(p+q)×1∑i=1n∑j=in(1p/(wiEiγ)+q/(wjEjγ)))1γ),1−1(1+(n(n+1)2(p+q)×1∑i=1n∑j=in(1p/(wiGiγ)+q/(wjGjγ)))1γ)])

*where* $B_{i}=\frac{b_{i}}{1-b_{i}},D_{i}=\frac{d_{i}}{1-d_{i}},E_{i}=\frac{1-e_{i}}{e_{i}},G_{i}=\frac{1-g_{i}}{g_{i}},B_{j}=\frac{b_{j}}{1-b_{j}},D_{j}=\frac{d_{j}}{1-d_{j}},E_{j}=\frac{1-e_{j}}{e_{j}},G_{j}=\frac{1-g_{j}}{g_{j}}$

**Proofs.**   

(35) $$\begin{array}{l} w_{i}{\tilde{\delta }}_{i}=\left(\left[1-\frac{1}{1+{\left(w_{i}{\left(\frac{b_{i}}{1-b_{i}}\right)}^{\gamma }\right)}^{\frac{1}{\gamma }}},1-\frac{1}{1+{\left(w_{i}{\left(\frac{d_{i}}{1-d_{i}}\right)}^{\gamma }\right)}^{\frac{1}{\gamma }}}\right],\left[\frac{1}{1+{\left(w_{i}{\left(\frac{1-e_{i}}{e_{i}}\right)}^{\gamma }\right)}^{\frac{1}{\gamma }}},\frac{1}{1+{\left(w_{i}{\left(\frac{1-g_{i}}{g_{i}}\right)}^{\gamma }\right)}^{\frac{1}{\gamma }}}\right]\right),\\ w_{j}{\tilde{\delta }}_{j}=\left(\left[1-\frac{1}{1+{\left(w_{j}{\left(\frac{b_{j}}{1-b_{j}}\right)}^{\gamma }\right)}^{\frac{1}{\gamma }}},1-\frac{1}{1+{\left(w_{j}{\left(\frac{d_{j}}{1-d_{j}}\right)}^{\gamma }\right)}^{\frac{1}{\gamma }}}\right],\left[\frac{1}{1+{\left(w_{j}{\left(\frac{1-e_{j}}{e_{j}}\right)}^{\gamma }\right)}^{\frac{1}{\gamma }}},\frac{1}{1+{\left(w_{j}{\left(\frac{1-g_{j}}{g_{j}}\right)}^{\gamma }\right)}^{\frac{1}{\gamma }}}\right]\right) \end{array}$$

Let $B_{i}=\frac{b_{i}}{1-b_{i}},D_{i}=\frac{d_{i}}{1-d_{i}},E_{i}=\frac{1-e_{i}}{e_{i}},G_{i}=\frac{1-g_{i}}{g_{i}},B_{j}=\frac{b_{j}}{1-b_{j}},D_{j}=\frac{d_{j}}{1-d_{j}},E_{j}=\frac{1-e_{j}}{e_{j}},G_{j}=\frac{1-g_{j}}{g_{j}}$,

Then,

(36) $$\begin{array}{l} {\left(w_{i}{\tilde{\delta }}_{i}\right)}^{p}=\left(\left[\frac{1}{1+{\left(p/\left(w_{i}B_{i}^{\gamma }\right)\right)}^{\frac{1}{\gamma }}},\frac{1}{1+{\left(p/\left(w_{i}D_{i}^{\gamma }\right)\right)}^{\frac{1}{\gamma }}}\right],\left[1-\frac{1}{1+{\left(p/\left(w_{i}E_{i}^{\gamma }\right)\right)}^{\frac{1}{\gamma }}},1-\frac{1}{1+{\left(p/\left(w_{i}G_{i}^{\gamma }\right)\right)}^{\frac{1}{\gamma }}}\right]\right),\\ {\left(w_{j}{\tilde{\delta }}_{j}\right)}^{q}=\left(\left[\frac{1}{1+{\left(q/\left(w_{j}B_{j}^{\gamma }\right)\right)}^{\frac{1}{\gamma }}},\frac{1}{1+{\left(q/\left(w_{j}D_{j}^{\gamma }\right)\right)}^{\frac{1}{\gamma }}}\right],\left[1-\frac{1}{1+{\left(q/\left(w_{j}E_{j}^{\gamma }\right)\right)}^{\frac{1}{\gamma }}},1-\frac{1}{1+{\left(q/\left(w_{j}G_{j}^{\gamma }\right)\right)}^{\frac{1}{\gamma }}}\right]\right). \end{array}$$

Thus,

(37) $${\left(w_{i}{\tilde{\delta }}_{i}\right)}^{p}⊗{\left(w_{j}{\tilde{\delta }}_{j}\right)}^{q}=\left(\begin{array}{l} \left[\frac{1}{1+{\left(p/\left(w_{i}B_{i}^{\gamma }\right)+q/\left(w_{j}B_{j}^{\gamma }\right)\right)}^{\frac{1}{\gamma }}},\frac{1}{1+{\left(p/\left(w_{i}D_{i}^{\gamma }\right)+q/\left(w_{j}D_{j}^{\gamma }\right)\right)}^{\frac{1}{\gamma }}}\right],\\ \left[1-\frac{1}{1+{\left(p/\left(w_{i}E_{i}^{\gamma }\right)+q/\left(w_{j}E_{j}^{\gamma }\right)\right)}^{\frac{1}{\gamma }}},1-\frac{1}{1+{\left(p/\left(w_{i}G_{i}^{\gamma }\right)+q/\left(w_{j}G_{j}^{\gamma }\right)\right)}^{\frac{1}{\gamma }}}\right] \end{array}\right)$$

Thereafter,

(38) $$\begin{array}{l} \overset{n}{\underset{i=1}{⊕}}\overset{n}{\underset{j=i}{⊕}}\left({\left(w_{i}{\tilde{\delta }}_{i}\right)}^{p}⊗{\left(w_{j}{\tilde{\delta }}_{j}\right)}^{q}\right)\\ =\left(\begin{array}{l} \left[1-\frac{1}{\left(1+{\left(\sum_{i=1}^{n}\sum_{j=i}^{n}\left(\frac{1}{p/\left(w_{i}B_{i}^{\gamma }\right)+q/\left(w_{j}B_{j}^{\gamma }\right)}\right)\right)}^{\frac{1}{\gamma }}\right)},1-\frac{1}{\left(1+{\left(\sum_{i=1}^{n}\sum_{j=i}^{n}\left(\frac{1}{p/\left(w_{i}D_{i}^{\gamma }\right)+q/\left(w_{j}D_{j}^{\gamma }\right)}\right)\right)}^{\frac{1}{\gamma }}\right)}\right],\\ \left[\frac{1}{\left(1+{\left(\sum_{i=1}^{n}\sum_{j=i}^{n}\left(\frac{1}{p/\left(w_{i}E_{i}^{\gamma }\right)+q/\left(w_{j}E_{j}^{\gamma }\right)}\right)\right)}^{\frac{1}{\gamma }}\right)},\frac{1}{\left(1+{\left(\sum_{i=1}^{n}\sum_{j=i}^{n}\left(\frac{1}{p/\left(w_{i}G_{i}^{\gamma }\right)+q/\left(w_{j}G_{j}^{\gamma }\right)}\right)\right)}^{\frac{1}{\gamma }}\right)}\right] \end{array}\right) \end{array}$$

Therefore,

(39) $$\begin{array}{l} \frac{2}{n(n+1)}\overset{n}{\underset{i=1}{⊕}}\overset{n}{\underset{j=i}{⊕}}\left({\left(w_{i}{\tilde{\delta }}_{i}\right)}^{p}⊗{\left(w_{j}{\tilde{\delta }}_{j}\right)}^{q}\right)\\ =\left(\begin{array}{l} \left[1-\frac{1}{\left(1+{\left(\frac{2}{n(n+1)}\sum_{i=1}^{n}\sum_{j=i}^{n}\left(\frac{1}{p/\left(w_{i}B_{i}^{\gamma }\right)+q/\left(w_{j}B_{j}^{\gamma }\right)}\right)\right)}^{\frac{1}{\gamma }}\right)},1-\frac{1}{\left(1+{\left(\frac{2}{n(n+1)}\sum_{i=1}^{n}\sum_{j=i}^{n}\left(\frac{1}{p/\left(w_{i}D_{i}^{\gamma }\right)+q/\left(w_{j}D_{j}^{\gamma }\right)}\right)\right)}^{\frac{1}{\gamma }}\right)}\right],\\ \left[\frac{1}{\left(1+{\left(\frac{2}{n(n+1)}\sum_{i=1}^{n}\sum_{j=i}^{n}\left(\frac{1}{p/\left(w_{i}E_{i}^{\gamma }\right)+q/\left(w_{j}E_{j}^{\gamma }\right)}\right)\right)}^{\frac{1}{\gamma }}\right)},\frac{1}{\left(1+{\left(\frac{2}{n(n+1)}\sum_{i=1}^{n}\sum_{j=i}^{n}\left(\frac{1}{p/\left(w_{i}G_{i}^{\gamma }\right)+q/\left(w_{j}G_{j}^{\gamma }\right)}\right)\right)}^{\frac{1}{\gamma }}\right)}\right] \end{array}\right) \end{array}$$

Furthermore,

(40) (2n(n+1)⊕i=1n⊕j=in((wiδ˜i)p⊗(wjδ˜j)q))1p+q=([1(1+(n(n+1)2(p+q)×1∑i=1n∑j=in(1p/(wiBiγ)+q/(wjBjγ)))1γ),1(1+(n(n+1)2(p+q)×1∑i=1n∑j=in(1p/(wiDiγ)+q/(wjDjγ)))1γ)],[1−1(1+(n(n+1)2(p+q)×1∑i=1n∑j=in(1p/(wiEiγ)+q/(wjEjγ)))1γ),1−1(1+(n(n+1)2(p+q)×1∑i=1n∑j=in(1p/(wiGiγ)+q/(wjGjγ)))1γ)])

Thus, (34) is right. □

**Example** **2.** *Let* ${\tilde{\delta }}_{1}=\left(\left[0.2,0.5\right],\left[0.3,0.5\right]\right),{\tilde{\delta }}_{2}=\left(\left[0.3,0.6\right],\left[0.1,0.3\right]\right)$ *, and* ${\tilde{\delta }}_{3}=\left(\left[0.1,0.2\right],\left[0.2,0.4\right]\right)$ *be three IVIFNs, and* p=2,q=1,γ=3,w=(0.6,0.3,0.1) *. Then we employ the IVIFDWHM operator to fuse three IVIFNs.*

*First,*

(41) $$\begin{array}{l} \sum_{i=1}^{n}\sum_{j=i}^{n}\left(\frac{1}{p/\left(w_{i}B_{i}^{\gamma }\right)+q/\left(w_{j}B_{j}^{\gamma }\right)}\right)=\sum_{i=1}^{3}\sum_{j=i}^{3}\left(\frac{1}{2/\left(w_{i}B_{i}^{3}\right)+1/\left(w_{j}B_{j}^{3}\right)}\right)\\ =\frac{1}{2/\left(w_{1}B_{1}^{3}\right)+1/\left(w_{1}B_{1}^{3}\right)}+\frac{1}{2/\left(w_{1}B_{1}^{3}\right)+1/\left(w_{2}B_{2}^{3}\right)}+\frac{1}{2/\left(w_{1}B_{1}^{3}\right)+1/\left(w_{3}B_{3}^{3}\right)}\\ +\frac{1}{2/\left(w_{2}B_{2}^{3}\right)+1/\left(w_{2}B_{2}^{3}\right)}+\frac{1}{2/\left(w_{2}B_{2}^{3}\right)+1/\left(w_{3}B_{3}^{3}\right)}+\frac{1}{2/\left(w_{3}B_{3}^{3}\right)+1/\left(w_{3}B_{3}^{3}\right)}\\ =\frac{1}{2/\left(0.6\times {\left(\frac{0.2}{1-0.2}\right)}^{3}\right)+1/\left(0.6\times {\left(\frac{0.2}{1-0.2}\right)}^{3}\right)}+\frac{1}{2/\left(0.6\times {\left(\frac{0.2}{1-0.2}\right)}^{3}\right)+1/\left(0.3\times {\left(\frac{0.3}{1-0.3}\right)}^{3}\right)}\\ +\frac{1}{2/\left(0.6\times {\left(\frac{0.2}{1-0.2}\right)}^{3}\right)+1/\left(0.1\times {\left(\frac{0.1}{1-0.1}\right)}^{3}\right)}+\frac{1}{2/\left(0.3\times {\left(\frac{0.3}{1-0.3}\right)}^{3}\right)+1/\left(0.3\times {\left(\frac{0.3}{1-0.3}\right)}^{3}\right)}\\ +\frac{1}{2/\left(0.3\times {\left(\frac{0.3}{1-0.3}\right)}^{3}\right)+1/\left(0.1\times {\left(\frac{0.1}{1-0.1}\right)}^{3}\right)}+\frac{1}{2/\left(0.1\times {\left(\frac{0.1}{1-0.1}\right)}^{3}\right)+1/\left(0.1\times {\left(\frac{0.1}{1-0.1}\right)}^{3}\right)}\\ =0.0152 \end{array}$$

*Then, we have:*

(42) 1(1+(n(n+1)2(p+q)×1∑i=1n∑j=in(1p/(wiBiγ)+q/(wjBjγ)))1γ)=1(1+(3(3+1)2(2+1)×1∑i=13∑j=i3(12/(wiBi3)+q/(wjBj3)))13)=0.1644

*Similarly, we have:*

(43) 1(1+(n(n+1)2(p+q)×1∑i=1n∑j=in(1p/(wiDiγ)+q/(wjDjγ)))1γ)=1(1+(3(3+1)2(2+1)×1∑i=13∑j=i3(12/(wiDi3)+q/(wjDj3)))13)=0.4214

*And,*

(44) 1−1(1+(n(n+1)2(p+q)×1∑i=1n∑j=in(1p/(wiEiγ)+q/(wjEjγ)))1γ)=1−1(1+(3(3+1)2(2+1)×1∑i=13∑j=i3(12/(wiEi3)+q/(wjEj3)))13)=0.2196

*And,*

(45) 1−1(1+(n(n+1)2(p+q)×1∑i=1n∑j=in(1p/(wiGiγ)+q/(wjGjγ)))1γ)=1−1(1+(3(3+1)2(2+1)×1∑i=13∑j=i3(12/(wiGi3)+q/(wjGj3)))13)=0.4881

*Finally,* ${\mathrm{IVIFWDHM}}_{w}^{2,1}({\tilde{\delta }}_{1},{\tilde{\delta }}_{2},{\tilde{\delta }}_{3})=\left(\left[0.1644,0.4214\right],\left[0.2196,0.4881\right]\right)$

*Then we list some good properties of IVIFWDHM operator.*

**Property** **4.** *(Monotonicity) Let* ${\tilde{\delta }}_{i}=\left(\left[b_{i},d_{i}\right],\left[e_{i},g_{i}\right]\right)(i=1,2,\ldots ,n)$ *and* ${\tilde{\theta }}_{i}=\left(\left[r_{i},h_{i}\right],\left[m_{i},f_{i}\right]\right)$ (i=1,2,…,n) *be two sets of IVIFNs. If* $b_{i}\leq r_{i},d_{i}\leq h_{i}\;and\;e_{i}\geq m_{i},g_{i}\geq f_{i}$ *hold for all* i *, then,*

(46) $${\mathrm{IVIFWDHM}}^{p,q}({\tilde{\delta }}_{1},{\tilde{\delta }}_{2},\ldots ,{\tilde{\delta }}_{n})\leq {\mathrm{IVIFWDHM}}^{p,q}({\tilde{\theta }}_{1},{\tilde{\theta }}_{2},\ldots ,{\tilde{\theta }}_{n})$$

*The proof is similar to Property 2 of IVIFDHM, therefore, it is omitted here.*

**Property** **5.** *(Boundedness) Let* ${\tilde{\delta }}_{i}=\left(\left[b_{i},d_{i}\right],\left[e_{i},g_{i}\right]\right)(i=1,2,\ldots ,n)$ *be a set of IVIFNs. If*

${\tilde{\delta }}_{\max}=\left(\left(\left[\max_{i}\left(b_{i}\right),\max_{i}\left(d_{i}\right)\right],\left[\min_{i}\left(e_{i}\right),\min_{i}\left(g_{i}\right)\right]\right)\right)$ *,*

${\tilde{\delta }}_{\min}=\left(\left[\min_{i}\left(b_{i}\right),\min_{i}\left(d_{i}\right)\right],\left[\max_{i}\left(e_{i}\right),\max_{i}\left(g_{i}\right)\right]\right)$

*and,* ${\tilde{\delta }}^{+}={\mathrm{IVIFWDHM}}^{p,q}({\tilde{\delta }}_{\max},{\tilde{\delta }}_{\max},\ldots ,{\tilde{\delta }}_{\max})$ *,* ${\tilde{\delta }}^{-}={\mathrm{IVIFWDHM}}^{p,q}({\tilde{\delta }}_{\min},{\tilde{\delta }}_{\min},\ldots ,{\tilde{\delta }}_{\min})$ *then,*

(47) $${\tilde{\delta }}^{-}\leq {\mathrm{IVIFWDHM}}^{p,q}({\tilde{\varphi }}_{1},{\tilde{\varphi }}_{2},\ldots ,{\tilde{\varphi }}_{n})\leq {\tilde{\delta }}^{+}$$

**Proofs.**   

Let ${\tilde{\delta }}^{+}=\left(\left(\left[b_{\max}^{+},d_{\max}^{+}\right],\left[e_{\min}^{+},g_{\min}^{+}\right]\right)\right)$, ${\tilde{\delta }}^{-}=\left(\left(\left[b_{\min}^{-},d_{\min}^{-}\right],\left[e_{\max}^{-},g_{\max}^{-}\right]\right)\right)$ and ${\mathrm{IVIFWDHM}}^{p,q}({\tilde{\delta }}_{1},{\tilde{\delta }}_{2},\ldots ,{\tilde{\delta }}_{n})={\tilde{\delta }}_{\alpha }=\left(\left[b_{\alpha },d_{\alpha }\right],\left[e_{\alpha },g_{\alpha }\right]\right)$, then according to Theorem 2, we can have:

(48) $$\begin{array}{l} B_{\max}=\frac{\max_{i}\left(b_{i}\right)}{1-\max_{i}\left(b_{i}\right)}\geq B_{i}=\frac{b_{i}}{1-b_{i}}\geq B_{\min}=\frac{\min_{i}\left(b_{i}\right)}{1-\min_{i}\left(b_{i}\right)},\\ E_{\min}=\frac{1-\min_{i}\left(e_{i}\right)}{\min_{i}\left(e_{i}\right)}\geq E_{i}=\frac{1-e_{i}}{e_{i}}\geq E_{\max}=\frac{1-\max_{i}\left(e_{i}\right)}{\max_{i}\left(e_{i}\right)} \end{array}$$

Then,

(49) bmax=1(1+(n(n+1)2(p+q)×1∑i=1n∑j=in(1p/(wiBmaxγ)+q/(wjBmaxγ)))1γ)≥bα=1(1+(n(n+1)2(p+q)×1∑i=1n∑j=in(1p/(wiBiγ)+q/(wjBjγ)))1γ)

Thus,

(50) $$b_{\alpha }\leq b_{\max}^{+}$$

Similarly, we have:

(51) $$b_{\min}^{-}\leq b_{\alpha },\;d_{\min}^{-}\leq d_{\alpha }\leq d_{\max}^{+},\;e_{\min}^{+}\leq e_{\alpha }\leq e_{\max}^{-},\;g_{\min}^{+}\leq g_{\alpha }\leq g_{\max}^{-}$$

So,

(52) $$\begin{array}{l} S\left(\tilde{\delta }-\right)=\frac{b_{\min}^{-}-e_{\max}^{-}+d_{\min}^{-}-g_{\max}^{-}}{2}\\ \leq S\left({\tilde{\delta }}_{\alpha }\right)=\frac{b_{\alpha }-e_{\alpha }+d_{\alpha }-g_{\alpha }}{2}\\ \leq S\left({\tilde{\delta }}^{+}\right)=\frac{b_{\max}^{+}-e_{\min}^{+}+d_{\max}^{+}-g_{\min}^{+}}{2} \end{array}$$

Thus, ${\tilde{\delta }}^{-}\leq {\mathrm{IVIFWDHM}}^{p,q}({\tilde{\delta }}_{1},{\tilde{\delta }}_{2},\ldots ,{\tilde{\delta }}_{n})={\tilde{\delta }}_{\alpha }\leq {\tilde{\delta }}^{+}$. Property 5 is proved. □

### 3.3. The IVIFDGHM Operator

Wu et al. [10] gave the geometric Heronian mean (GHM) operator.

**Definition** **12** [10]**.** *The GHM operator has the form:*

(53) $$GHM^{p,q}\left(\delta_{1},\delta_{2},\cdots ,\delta_{n}\right)={\left(\frac{1}{p+q}\prod_{i=1}^{n}\prod_{j=i}^{n}p\delta_{i}+q\delta_{j}\right)}^{\frac{2}{n(n+1)}}$$

*where p, q ≥ 0, then* $\delta_{i}\left(i=1,2,\cdots ,n\right)$ *is a series of crisp numbers.*

Based on the GHM operator, we develop the interval-valued intuitionistic fuzzy Dombi GHM (IVIFDGHM) operator.

**Definition** **13.** *Let* ${\tilde{\delta }}_{i}=\left(\left[b_{i},d_{i}\right],\left[e_{i},g_{i}\right]\right)\left(i=1,2,\ldots ,n\right)$ *be a set of IVIFNs. The IVIFDGHM operator is:*

(54) $${\mathrm{IVIFDGHM}}^{p,q}\left({\tilde{\delta }}_{1},{\tilde{\delta }}_{2},\cdots ,{\tilde{\delta }}_{n}\right)=\frac{1}{p+q}\overset{n}{\underset{i=1}{⊗}}\overset{n}{\underset{j=i}{⊗}}\left({\left(p{\tilde{\delta }}_{i}⊕q{\tilde{\delta }}_{j}\right)}^{\frac{2}{n(n+1)}}\right)$$

**Theorem** **3.** *Let* ${\tilde{\delta }}_{i}=\left(\left[b_{i},d_{i}\right],\left[e_{i},g_{i}\right]\right)\left(i=1,2,\ldots ,n\right)$ *be a set of IVIFNs and* p,q≥0,γ>0 *. The fused value by IVIFDGHM operators is also an IVIFN, where:*

(55) IVIFDGHMp,q(δ˜1,δ˜2,⋯,δ˜n)=1p+q⊗i=1n⊗j=in((pδ˜i⊕qδ˜j)2n(n+1))=([1−1(1+(n(n+1)2(p+q)×(1∑i=1n∑j=in1pBiγ+qBjγ))1γ),1−1(1+(n(n+1)2(p+q)×(1∑i=1n∑j=in1pDiγ+qDjγ))1γ)],[1(1+(n(n+1)2(p+q)×(1∑i=1n∑j=in1pEiγ+qEjγ))1γ),1(1+(n(n+1)2(p+q)×(1∑i=1n∑j=in1pGiγ+qGjγ))1γ)])

*where* $B_{i}=\frac{b_{i}}{1-b_{i}},D_{i}=\frac{d_{i}}{1-d_{i}},E_{i}=\frac{1-e_{i}}{e_{i}},G_{i}=\frac{1-g_{i}}{g_{i}},B_{j}=\frac{b_{j}}{1-b_{j}},D_{j}=\frac{d_{j}}{1-d_{j}},E_{j}=\frac{1-e_{j}}{e_{j}},G_{j}=\frac{1-g_{j}}{g_{j}}$

**Proofs.**   

(56) $$\begin{array}{l} p{\tilde{\delta }}_{i}=\left(\left[1-\frac{1}{1+{\left(p{\left(\frac{b_{i}}{1-b_{i}}\right)}^{\gamma }\right)}^{\frac{1}{\gamma }}},1-\frac{1}{1+{\left(p{\left(\frac{d_{i}}{1-d_{i}}\right)}^{\gamma }\right)}^{\frac{1}{\gamma }}}\right],\left[\frac{1}{1+{\left(p{\left(\frac{1-e_{i}}{e_{i}}\right)}^{\gamma }\right)}^{\frac{1}{\gamma }}},\frac{1}{1+{\left(p{\left(\frac{1-g_{i}}{g_{i}}\right)}^{\gamma }\right)}^{\frac{1}{\gamma }}}\right]\right),\\ q{\tilde{\delta }}_{j}=\left(\left[1-\frac{1}{1+{\left(q{\left(\frac{b_{j}}{1-b_{j}}\right)}^{\gamma }\right)}^{\frac{1}{\gamma }}},1-\frac{1}{1+{\left(q{\left(\frac{d_{j}}{1-d_{j}}\right)}^{\gamma }\right)}^{\frac{1}{\gamma }}}\right],\left[\frac{1}{1+{\left(q{\left(\frac{1-e_{j}}{e_{j}}\right)}^{\gamma }\right)}^{\frac{1}{\gamma }}},\frac{1}{1+{\left(q{\left(\frac{1-g_{j}}{g_{j}}\right)}^{\gamma }\right)}^{\frac{1}{\gamma }}}\right]\right) \end{array}$$

Let $B_{i}=\frac{b_{i}}{1-b_{i}},D_{i}=\frac{d_{i}}{1-d_{i}},E_{i}=\frac{1-e_{i}}{e_{i}},G_{i}=\frac{1-g_{i}}{g_{i}},B_{j}=\frac{b_{j}}{1-b_{j}},D_{j}=\frac{d_{j}}{1-d_{j}},E_{j}=\frac{1-e_{j}}{e_{j}},G_{j}=\frac{1-g_{j}}{g_{j}}$,

Then,

(57) $$\begin{array}{l} p{\tilde{\delta }}_{i}=\left(\left[1-\frac{1}{1+{\left(pB_{i}^{\gamma }\right)}^{\frac{1}{\gamma }}},1-\frac{1}{1+{\left(pD_{i}^{\gamma }\right)}^{\frac{1}{\gamma }}}\right],\left[\frac{1}{1+{\left(pE_{i}^{\gamma }\right)}^{\frac{1}{\gamma }}},\frac{1}{1+{\left(pG_{i}^{\gamma }\right)}^{\frac{1}{\gamma }}}\right]\right),\\ q{\tilde{\delta }}_{j}=\left(\left[1-\frac{1}{1+{\left(qB_{j}^{\gamma }\right)}^{\frac{1}{\gamma }}},1-\frac{1}{1+{\left(qD_{j}^{\gamma }\right)}^{\frac{1}{\gamma }}}\right],\left[\frac{1}{1+{\left(qE_{j}^{\gamma }\right)}^{\frac{1}{\gamma }}},\frac{1}{1+{\left(qG_{j}^{\gamma }\right)}^{\frac{1}{\gamma }}}\right]\right) \end{array}$$

Thus,

(58) $$p{\tilde{\delta }}_{i}⊕q{\tilde{\delta }}_{j}=\left(\begin{array}{l} \left[1-\frac{1}{1+{\left(pB_{i}^{\gamma }+qB_{j}^{\gamma }\right)}^{\frac{1}{\gamma }}},1-\frac{1}{1+{\left(pD_{i}^{\gamma }+qD_{j}^{\gamma }\right)}^{\frac{1}{\gamma }}}\right],\\ \left[\frac{1}{1+{\left(pE_{i}^{\gamma }+qE_{j}^{\gamma }\right)}^{\frac{1}{\gamma }}},\frac{1}{1+{\left(pG_{i}^{\lambda }+qG_{j}^{\gamma }\right)}^{\frac{1}{\gamma }}}\right] \end{array}\right)$$

Thereafter,

(59) $${\left(p{\tilde{\delta }}_{i}⊕q{\tilde{\delta }}_{j}\right)}^{\frac{2}{n(n+1)}}=\left(\begin{array}{l} \left[\frac{1}{1+{\left(\frac{2}{n(n+1)}\times \frac{1}{pB_{i}^{\gamma }+qB_{j}^{\gamma }}\right)}^{\frac{1}{\gamma }}},\frac{1}{1+{\left(\frac{2}{n(n+1)}\times \frac{1}{pD_{i}^{\gamma }+qD_{j}^{\gamma }}\right)}^{\frac{1}{\gamma }}}\right],\\ \left[1-\frac{1}{1+{\left(\frac{2}{n(n+1)}\times \frac{1}{pE_{i}^{\gamma }+qE_{j}^{\gamma }}\right)}^{\frac{1}{\gamma }}},1-\frac{1}{1+{\left(\frac{2}{n(n+1)}\times \frac{1}{pG_{i}^{\gamma }+qG_{j}^{\gamma }}\right)}^{\frac{1}{\gamma }}}\right] \end{array}\right)$$

And,

(60) $$\overset{n}{\underset{j=i}{⊗}}\left({\left(p{\tilde{\delta }}_{i}⊕q{\tilde{\delta }}_{j}\right)}^{\frac{2}{n(n+1)}}\right)=\left(\begin{array}{l} \left[\frac{1}{1+{\left(\frac{2}{n(n+1)}\times \sum_{j=i}^{n}\frac{1}{pB_{i}^{\gamma }+qB_{j}^{\gamma }}\right)}^{\frac{1}{\gamma }}},\frac{1}{1+{\left(\frac{2}{n(n+1)}\times \sum_{j=i}^{n}\frac{1}{pD_{i}^{\gamma }+qD_{j}^{\gamma }}\right)}^{\frac{1}{\gamma }}}\right],\\ \left[1-\frac{1}{1+{\left(\frac{2}{n(n+1)}\times \sum_{j=i}^{n}\frac{1}{pE_{i}^{\gamma }+qE_{j}^{\gamma }}\right)}^{\frac{1}{\gamma }}},1-\frac{1}{1+{\left(\frac{2}{n(n+1)}\times \sum_{j=i}^{n}\frac{1}{pG_{i}^{\gamma }+qG_{j}^{\gamma }}\right)}^{\frac{1}{\gamma }}}\right] \end{array}\right)$$

Therefore,

(61) $$\begin{array}{l} \overset{n}{\underset{i=1}{⊗}}\overset{n}{\underset{j=i}{⊗}}\left({\left(p{\tilde{\delta }}_{i}⊕q{\tilde{\delta }}_{j}\right)}^{\frac{2}{n(n+1)}}\right)\\ =\left(\begin{array}{l} \left[\frac{1}{\left(1+{\left(\frac{2}{n(n+1)}\times \sum_{i=1}^{n}\sum_{j=i}^{n}\frac{1}{pB_{i}^{\gamma }+qB_{j}^{\gamma }}\right)}^{\frac{1}{\gamma }}\right)},\frac{1}{\left(1+{\left(\frac{2}{n(n+1)}\times \sum_{i=1}^{n}\sum_{j=i}^{n}\frac{1}{pD_{i}^{\gamma }+qD_{j}^{\gamma }}\right)}^{\frac{1}{\gamma }}\right)}\right],\\ \left[1-\frac{1}{\left(1+{\left(\frac{2}{n(n+1)}\times \sum_{i=1}^{n}\sum_{j=i}^{n}\frac{1}{pE_{i}^{\gamma }+qE_{j}^{\gamma }}\right)}^{\frac{1}{\gamma }}\right)},1-\frac{1}{\left(1+{\left(\frac{2}{n(n+1)}\times \sum_{i=1}^{n}\sum_{j=i}^{n}\frac{1}{pG_{i}^{\gamma }+qG_{j}^{\gamma }}\right)}^{\frac{1}{\gamma }}\right)}\right] \end{array}\right) \end{array}$$

Furthermore,

(62) 1p+q⊗i=1n⊗j=in((pδ˜i⊕qδ˜j)2n(n+1))=([1−1(1+(n(n+1)2(p+q)×(1∑i=1n∑j=in1pBiγ+qBjγ))1γ),1−1(1+(n(n+1)2(p+q)×(1∑i=1n∑j=in1pDiγ+qDjγ))1γ)],[1(1+(n(n+1)2(p+q)×(1∑i=1n∑j=in1pEiγ+qEjγ))1γ),1(1+(n(n+1)2(p+q)×(1∑i=1n∑j=in1pGiγ+qGjγ))1γ)])

Thus, (55) is right. □

**Example** **3.** *Let* ${\tilde{\delta }}_{1}=\left(\left[0.2,0.5\right],\left[0.3,0.5\right]\right),{\tilde{\delta }}_{2}=\left(\left[0.3,0.6\right],\left[0.1,0.3\right]\right)$ *, and* ${\tilde{\delta }}_{3}=\left(\left[0.1,0.2\right],\left[0.2,0.4\right]\right)$ *be three IVIFNs, and,* p=2,q=1,γ=3 *. Then we employ the IVIFDGHM operator to fuse three IVIFNs.*

*First,*

(63) $$\begin{array}{l} \sum_{i=1}^{n}\sum_{j=i}^{n}\frac{1}{pB_{i}^{\gamma }+qB_{j}^{\gamma }}=\sum_{i=1}^{3}\sum_{j=i}^{3}\frac{1}{2\times B_{i}^{3}+1\times B_{j}^{3}},\\ =\frac{1}{2\times B_{1}^{3}+1\times B_{1}^{3}}+\frac{1}{2\times B_{1}^{3}+1\times B_{2}^{3}}+\frac{1}{2\times B_{1}^{3}+1\times B_{3}^{3}},\\ +\frac{1}{2\times B_{2}^{3}+1\times B_{2}^{3}}+\frac{1}{2\times B_{2}^{3}+1\times B_{3}^{3}}+\frac{1}{2\times B_{3}^{3}+1\times B_{3}^{3}},\\ =\frac{1}{2\times {\left(\frac{0.2}{1-0.2}\right)}^{3}+1\times {\left(\frac{0.2}{1-0.2}\right)}^{3}}+\frac{1}{2\times {\left(\frac{0.2}{1-0.2}\right)}^{3}+1\times {\left(\frac{0.3}{1-0.3}\right)}^{3}}+\frac{1}{2\times {\left(\frac{0.2}{1-0.2}\right)}^{3}+1\times {\left(\frac{0.1}{1-0.1}\right)}^{3}},\\ +\frac{1}{2\times {\left(\frac{0.3}{1-0.3}\right)}^{3}+1\times {\left(\frac{0.3}{1-0.3}\right)}^{3}}+\frac{1}{2\times {\left(\frac{0.3}{1-0.3}\right)}^{3}+1\times {\left(\frac{0.1}{1-0.1}\right)}^{3}}+\frac{1}{2\times {\left(\frac{0.1}{1-0.1}\right)}^{3}+1\times {\left(\frac{0.1}{1-0.1}\right)}^{3}},\\ =314.6129 \end{array}$$

*Then, we have:*

(64) 1−1(1+(n(n+1)2(p+q)×(1∑i=1n∑j=in1pBiγ+qBjγ))1γ)=1−1(1+(3(3+1)2(2+1)×(1∑i=13∑j=i312×Bi3+1×Bj3))13)=0.1563

*Similarly, we have:*

(65) 1−1(1+(n(n+1)2(p+q)×(1∑i=1n∑j=in1pDiγ+qDjγ))1γ)=1−1(1+(3(3+1)2(2+1)×(1∑i=1n∑j=in12×Di3+1×Dj3))13)=0.3083

*And,*

(66) 1(1+(n(n+1)2(p+q)×(1∑i=1n∑j=in1pEiγ+qEjγ))1γ)=1(1+(3(3+1)2(2+1)×(1∑i=1n∑j=in12×Ei3+1×Ej3))13)=0.2202

*And,*

(67) 1(1+(n(n+1)2(p+q)×(1∑i=1n∑j=in1pGiγ+qGjγ))1γ)=1(1+(3(3+1)2(2+1)×(1∑i=13∑j=i312×Gi3+1×Gj3))13)=0.4187

*Finally,* ${\mathrm{IVIFDGHM}}^{2,1}({\tilde{\delta }}_{1},{\tilde{\delta }}_{2},{\tilde{\delta }}_{3})=\left(\left[0.1563,0.3083\right],\left[0.2202,0.4187\right]\right)$

*The IVIFDGHM operator also has the following properties. The proof is similar to IVIFDHM.*

**Property** **6.** *(Idempotency) If* ${\tilde{\delta }}_{i}=\left(\left[b_{i},d_{i}\right],\left[e_{i},g_{i}\right]\right)(i=1,2,\ldots ,n)$ *are equal, then*

(68) $${\mathrm{IVIFDGHM}}^{p,q}\left({\tilde{\delta }}_{1},{\tilde{\delta }}_{2},\cdots ,{\tilde{\delta }}_{n}\right)=\tilde{\delta }$$

**Property** **7.** *(Monotonicity) Let* ${\tilde{\delta }}_{i}=\left(\left[b_{i},d_{i}\right],\left[e_{i},g_{i}\right]\right)(i=1,2,\ldots ,n)$ *, and* ${\tilde{\theta }}_{i}=\left(\left[r_{i},h_{i}\right],\left[m_{i},f_{i}\right]\right)$ (i=1,2,…,n) *be two sets of IVIFNs. If* $b_{i}\leq r_{i},d_{i}\leq h_{i}\;and\;e_{i}\geq m_{i},g_{i}\geq f_{i}$ *hold for all* i *, then,*

(69) $${\mathrm{IVIFDGHM}}^{p,q}\left({\tilde{\delta }}_{1},{\tilde{\delta }}_{2},\cdots ,{\tilde{\delta }}_{n}\right)\leq {\mathrm{IVIFDGHM}}^{p,q}\left({\tilde{\theta }}_{1},{\tilde{\theta }}_{2},\cdots ,{\tilde{\theta }}_{n}\right)$$

**Property** **8.** *(Boundedness) Let* ${\tilde{\delta }}_{i}=\left(\left[b_{i},d_{i}\right],\left[e_{i},g_{i}\right]\right)(i=1,2,\ldots ,n)$ *be a set of IVIFNs. If* ${\tilde{\delta }}^{+}=\left(\left(\left[\max_{i}\left(b_{i}\right),\max_{i}\left(d_{i}\right)\right],\left[\min_{i}\left(e_{i}\right),\min_{i}\left(g_{i}\right)\right]\right)\right)$ *and* ${\tilde{\delta }}^{-}=\left(\left[\min_{i}\left(b_{i}\right),\min_{i}\left(d_{i}\right)\right],\left[\max_{i}\left(e_{i}\right),\max_{i}\left(g_{i}\right)\right]\right)$ *, then,*

(70) $${\tilde{\delta }}^{-}\leq {\mathrm{IVIFDGHM}}^{p,q}\left({\tilde{\delta }}_{1},{\tilde{\delta }}_{2},\cdots ,{\tilde{\delta }}_{n}\right)\leq {\tilde{\delta }}^{+}$$

### 3.4. The IVIFWDGHM Operator

In some practical MADM, it’s very important to pay attention to attribute weights; we define the interval-valued intuitionistic weighted Dombi GHM (IVIFWDGHM) operator.

**Definition** **14.** *Let*${\tilde{\delta }}_{i}=\left(\left[b_{i},d_{i}\right],\left[e_{i},g_{i}\right]\right)(i=1,2,\ldots ,n)$*be a set of IVIFNs with their weight vector be*$w_{i}={\left(w_{1},w_{2},\ldots ,w_{n}\right)}^{T}$*, thereby satisfying*$w_{i}\in \left[0,1\right]$*and*$\sum_{i=1}^{n}w_{i}=1$.

(71) $${\mathrm{IVIFWDGHM}}_{w}^{p,q}\left({\tilde{\delta }}_{1},{\tilde{\delta }}_{2},\cdots ,{\tilde{\delta }}_{n}\right)=\frac{1}{p+q}\overset{n}{\underset{i=1}{⊗}}\overset{n}{\underset{j=i}{⊗}}\left({\left(p{\left({\tilde{\delta }}_{i}\right)}^{w_{i}}⊕q{\left({\tilde{\delta }}_{j}\right)}^{w_{j}}\right)}^{\frac{2}{n(n+1)}}\right)$$

**Theorem** **4.** *Let* ${\tilde{\delta }}_{i}=\left(\left[b_{i},d_{i}\right],\left[e_{i},g_{i}\right]\right)(i=1,2,\ldots ,n)$ *be a set of IVIFNs and* p,q≥0,γ>0 *. The fused value by IVIFWDGHM operators is also an IVIFN, where:*

${\mathrm{IVIFWDGHM}}_{w}^{p,q}\left({\tilde{\delta }}_{1},{\tilde{\delta }}_{2},\cdots ,{\tilde{\delta }}_{n}\right)=\frac{1}{p+q}\overset{n}{\underset{i=1}{⊗}}\overset{n}{\underset{j=i}{⊗}}\left({\left(p{\left({\tilde{\delta }}_{i}\right)}^{w_{i}}⊕q{\left({\tilde{\delta }}_{j}\right)}^{w_{j}}\right)}^{\frac{2}{n(n+1)}}\right)$

(72) =([1−1(1+(n(n+1)2(p+q)×1∑i=1n∑j=in(1p/(wiBiγ)+q/(wjBjγ)))1γ),1−1(1+(n(n+1)2(p+q)×1∑i=1n∑j=in(1p/(wiDiγ)+q/(wjDjγ)))1γ)],[1(1+(n(n+1)2(p+q)×1∑i=1n∑j=in(1p/(wiEiγ)+q/(wjEjγ)))1γ),1(1+(n(n+1)2(p+q)×1∑i=1n∑j=in(1p/(wiGiγ)+q/(wjGjγ)))1γ)])

*where* $B_{i}=\frac{1-b_{i}}{b_{i}},D_{i}=\frac{1-d_{i}}{d_{i}},E_{i}=\frac{e_{i}}{1-e_{i}},G_{i}=\frac{g_{i}}{1-g_{i}},B_{j}=\frac{1-b_{j}}{b_{j}},D_{j}=\frac{1-d_{j}}{d_{j}},E_{j}=\frac{e_{j}}{1-e_{j}},G_{j}=\frac{g_{j}}{1-g_{j}}$

**Proofs.**   

(73) $$\begin{array}{l} {\left({\tilde{\delta }}_{i}\right)}^{w_{i}}=\left(\left[\frac{1}{1+{\left(w_{i}{\left(\frac{1-b_{i}}{b_{i}}\right)}^{\gamma }\right)}^{\frac{1}{\gamma }}},\frac{1}{1+{\left(w_{i}{\left(\frac{1-d_{i}}{d_{i}}\right)}^{\gamma }\right)}^{\frac{1}{\gamma }}}\right],\left[1-\frac{1}{1+{\left(w_{i}{\left(\frac{e_{i}}{1-e_{i}}\right)}^{\gamma }\right)}^{\frac{1}{\gamma }}},1-\frac{1}{1+{\left(w_{i}{\left(\frac{g_{i}}{1-g_{i}}\right)}^{\gamma }\right)}^{\frac{1}{\gamma }}}\right]\right),\\ {\left({\tilde{\delta }}_{j}\right)}^{w_{j}}=\left(\left[\frac{1}{1+{\left(w_{j}{\left(\frac{1-b_{j}}{b_{j}}\right)}^{\gamma }\right)}^{\frac{1}{\gamma }}},\frac{1}{1+{\left(w_{j}{\left(\frac{1-d_{j}}{d_{j}}\right)}^{\gamma }\right)}^{\frac{1}{\gamma }}}\right],\left[1-\frac{1}{1+{\left(w_{j}{\left(\frac{e_{j}}{1-e_{j}}\right)}^{\gamma }\right)}^{\frac{1}{\gamma }}},1-\frac{1}{1+{\left(w_{j}{\left(\frac{g_{j}}{1-g_{j}}\right)}^{\gamma }\right)}^{\frac{1}{\gamma }}}\right]\right). \end{array}$$

Let $B_{i}=\frac{1-b_{i}}{b_{i}},D_{i}=\frac{1-d_{i}}{d_{i}},E_{i}=\frac{e_{i}}{1-e_{i}},G_{i}=\frac{g_{i}}{1-g_{i}},B_{j}=\frac{1-b_{j}}{b_{j}},D_{j}=\frac{1-d_{j}}{d_{j}},E_{j}=\frac{e_{j}}{1-e_{j}},G_{j}=\frac{g_{j}}{1-g_{j}}$,

Then,

(74) $$\begin{array}{l} p{\left({\tilde{\delta }}_{i}\right)}^{w_{i}}=\left(\left[1-\frac{1}{1+{\left(p/\left(w_{i}B_{i}^{\gamma }\right)\right)}^{\frac{1}{\gamma }}},1-\frac{1}{1+{\left(p/\left(w_{i}D_{i}^{\gamma }\right)\right)}^{\frac{1}{\gamma }}}\right],\left[\frac{1}{1+{\left(p/\left(w_{i}E_{i}^{\gamma }\right)\right)}^{\frac{1}{\gamma }}},\frac{1}{1+{\left(p/\left(w_{i}G_{i}^{\gamma }\right)\right)}^{\frac{1}{\gamma }}}\right]\right)\\ q{\left({\tilde{\delta }}_{j}\right)}^{w_{j}}=\left(\left[1-\frac{1}{1+{\left(q/\left(w_{j}B_{j}^{\gamma }\right)\right)}^{\frac{1}{\gamma }}},1-\frac{1}{1+{\left(q/\left(w_{j}D_{j}^{\gamma }\right)\right)}^{\frac{1}{\gamma }}}\right],\left[\frac{1}{1+{\left(q/\left(w_{j}E_{j}^{\gamma }\right)\right)}^{\frac{1}{\gamma }}},\frac{1}{1+{\left(q/\left(w_{j}G_{j}^{\gamma }\right)\right)}^{\frac{1}{\gamma }}}\right]\right) \end{array}$$

Thus,

(75) $$p{\left({\tilde{\delta }}_{i}\right)}^{w_{i}}⊕q{\left({\tilde{\delta }}_{j}\right)}^{w_{j}}=\left(\begin{array}{l} \left[1-\frac{1}{1+{\left(p/\left(w_{i}B_{i}^{\gamma }\right)+q/\left(w_{j}B_{j}^{\gamma }\right)\right)}^{\frac{1}{\gamma }}},1-\frac{1}{1+{\left(p/\left(w_{i}D_{i}^{\gamma }\right)+q/\left(w_{j}D_{j}^{\gamma }\right)\right)}^{\frac{1}{\gamma }}}\right],\\ \left[\frac{1}{1+{\left(p/\left(w_{i}E_{i}^{\gamma }\right)+q/\left(w_{j}E_{j}^{\gamma }\right)\right)}^{\frac{1}{\gamma }}},\frac{1}{1+{\left(p/\left(w_{i}G_{i}^{\gamma }\right)+q/\left(w_{j}G_{j}^{\gamma }\right)\right)}^{\frac{1}{\gamma }}}\right] \end{array}\right)$$

Thereafter,

(76) $$\begin{array}{l} {\left(p{\left({\tilde{\delta }}_{i}\right)}^{w_{i}}⊕q{\left({\tilde{\delta }}_{j}\right)}^{w_{j}}\right)}^{\frac{2}{n(n+1)}}\\ =\left(\begin{array}{l} \left[\frac{1}{\left(1+{\left(\frac{2}{n(n+1)}\times \frac{1}{p/\left(w_{i}B_{i}^{\gamma }\right)+q/\left(w_{j}B_{j}^{\gamma }\right)}\right)}^{\frac{1}{\gamma }}\right)},\frac{1}{\left(1+{\left(\frac{2}{n(n+1)}\times \frac{1}{p/\left(w_{i}D_{i}^{\gamma }\right)+q/\left(w_{j}D_{j}^{\gamma }\right)}\right)}^{\frac{1}{\gamma }}\right)}\right],\\ \left[1-\frac{1}{\left(1+{\left(\frac{2}{n(n+1)}\times \frac{1}{p/\left(w_{i}E_{i}^{\gamma }\right)+q/\left(w_{j}E_{j}^{\gamma }\right)}\right)}^{\frac{1}{\gamma }}\right)},1-\frac{1}{\left(1+{\left(\frac{2}{n(n+1)}\times \frac{1}{p/\left(w_{i}G_{i}^{\gamma }\right)+q/\left(w_{j}G_{j}^{\gamma }\right)}\right)}^{\frac{1}{\gamma }}\right)}\right] \end{array}\right) \end{array}$$

Therefore,

(77) $$\begin{array}{l} \overset{n}{\underset{i=1}{⊗}}\overset{n}{\underset{j=i}{⊗}}\left({\left(p{\left({\tilde{\delta }}_{i}\right)}^{w_{i}}⊕q{\left({\tilde{\delta }}_{j}\right)}^{w_{j}}\right)}^{\frac{2}{n(n+1)}}\right)\\ =\left(\begin{array}{l} \left[\frac{1}{\left(1+{\left(\frac{2}{n(n+1)}\sum_{i=1}^{n}\sum_{j=i}^{n}\left(\frac{1}{p/\left(w_{i}B_{i}^{\gamma }\right)+q/\left(w_{j}B_{j}^{\gamma }\right)}\right)\right)}^{\frac{1}{\gamma }}\right)},\frac{1}{\left(1+{\left(\frac{2}{n(n+1)}\sum_{i=1}^{n}\sum_{j=i}^{n}\left(\frac{1}{p/\left(w_{i}D_{i}^{\gamma }\right)+q/\left(w_{j}D_{j}^{\gamma }\right)}\right)\right)}^{\frac{1}{\gamma }}\right)}\right],\\ \left[1-\frac{1}{\left(1+{\left(\frac{2}{n(n+1)}\sum_{i=1}^{n}\sum_{j=i}^{n}\left(\frac{1}{p/\left(w_{i}E_{i}^{\gamma }\right)+q/\left(w_{j}E_{j}^{\gamma }\right)}\right)\right)}^{\frac{1}{\gamma }}\right)},1-\frac{1}{\left(1+{\left(\frac{2}{n(n+1)}\sum_{i=1}^{n}\sum_{j=i}^{n}\left(\frac{1}{p/\left(w_{i}G_{i}^{\gamma }\right)+q/\left(w_{j}G_{j}^{\gamma }\right)}\right)\right)}^{\frac{1}{\gamma }}\right)}\right] \end{array}\right) \end{array}$$

Furthermore,

(78) 1p+q⊗i=1n⊗j=in((p(δ˜i)wi⊕q(δ˜j)wj)2n(n+1))=([1−1(1+(n(n+1)2(p+q)×1∑i=1n∑j=in(1p/(wiBiγ)+q/(wjBjγ)))1γ),1−1(1+(n(n+1)2(p+q)×1∑i=1n∑j=in(1p/(wiDiγ)+q/(wjDjγ)))1γ)],[1(1+(n(n+1)2(p+q)×1∑i=1n∑j=in(1p/(wiEiγ)+q/(wjEjγ)))1γ),1(1+(n(n+1)2(p+q)×1∑i=1n∑j=in(1p/(wiGiγ)+q/(wjGjγ)))1γ)])

Thus, (72) is right. □

**Example** **4.** *Let* ${\tilde{\delta }}_{1}=\left(\left[0.2,0.5\right],\left[0.3,0.5\right]\right),{\tilde{\delta }}_{2}=\left(\left[0.3,0.6\right],\left[0.1,0.3\right]\right)$ *, and* ${\tilde{\delta }}_{3}=\left(\left[0.1,0.2\right],\left[0.2,0.4\right]\right)$ *be three IVIFNs, and,* p=2,q=1,γ=3,w=(0.6,0.3,0.1) *. Then we employ the IVIFDWGHM operator to fuse three IVIFNs.*

*First,*

(79) $$\begin{array}{l} \sum_{i=1}^{n}\sum_{j=i}^{n}\left(\frac{1}{p/\left(w_{i}B_{i}^{\gamma }\right)+q/\left(w_{j}B_{j}^{\gamma }\right)}\right)=\sum_{i=1}^{3}\sum_{j=i}^{3}\left(\frac{1}{2/\left(w_{i}B_{i}^{3}\right)+1/\left(w_{j}B_{j}^{3}\right)}\right)\\ =\frac{1}{2/\left(w_{1}B_{1}^{3}\right)+1/\left(w_{1}B_{1}^{3}\right)}+\frac{1}{2/\left(w_{1}B_{1}^{3}\right)+1/\left(w_{2}B_{2}^{3}\right)}+\frac{1}{2/\left(w_{1}B_{1}^{3}\right)+1/\left(w_{3}B_{3}^{3}\right)}\\ +\frac{1}{2/\left(w_{2}B_{2}^{3}\right)+1/\left(w_{2}B_{2}^{3}\right)}+\frac{1}{2/\left(w_{2}B_{2}^{3}\right)+1/\left(w_{3}B_{3}^{3}\right)}+\frac{1}{2/\left(w_{3}B_{3}^{3}\right)+1/\left(w_{3}B_{3}^{3}\right)}\\ =\frac{1}{2/\left(0.6\times {\left(\frac{1-0.2}{0.2}\right)}^{3}\right)+1/\left(0.6\times {\left(\frac{1-0.2}{0.2}\right)}^{3}\right)}+\frac{1}{2/\left(0.6\times {\left(\frac{1-0.2}{0.2}\right)}^{3}\right)+1/\left(0.3\times {\left(\frac{1-0.3}{0.3}\right)}^{3}\right)}\\ +\frac{1}{2/\left(0.6\times {\left(\frac{1-0.2}{0.2}\right)}^{3}\right)+1/\left(0.1\times {\left(\frac{1-0.1}{0.1}\right)}^{3}\right)}+\frac{1}{2/\left(0.3\times {\left(\frac{1-0.3}{0.3}\right)}^{3}\right)+1/\left(0.3\times {\left(\frac{1-0.3}{0.3}\right)}^{3}\right)}\\ +\frac{1}{2/\left(0.3\times {\left(\frac{1-0.3}{0.3}\right)}^{3}\right)+1/\left(0.1\times {\left(\frac{1-0.1}{0.1}\right)}^{3}\right)}+\frac{1}{2/\left(0.1\times {\left(\frac{1-0.1}{0.1}\right)}^{3}\right)+1/\left(0.1\times {\left(\frac{1-0.1}{0.1}\right)}^{3}\right)}\\ =58.6074 \end{array}$$

*Then, we have:*

(80) 1−1(1+(n(n+1)2(p+q)×1∑i=1n∑j=in(1p/(wiBiγ)+q/(wjBjγ)))1γ)=1−1(1+(3(3+1)2(2+1)×1∑i=13∑j=i3(12/(wiBi3)+q/(wjBj3)))13)=0.2449

*Similarly, we have:*

(81) 1−1(1+(n(n+1)2(p+q)×1∑i=1n∑j=in(1p/(wiDiγ)+q/(wjDjγ)))1γ)=1−1(1+(3(3+1)2(2+1)×1∑i=13∑j=i3(12/(wiDi3)+q/(wjDj3)))13)=0.4731

*And,*

(82) 1(1+(n(n+1)2(p+q)×1∑i=1n∑j=in(1p/(wiEiγ)+q/(wjEjγ)))1γ)=1(1+(3(3+1)2(2+1)×1∑i=13∑j=i3(12/(wiEi3)+q/(wjEj3)))13)=0.1734

*And,*

(83) 1(1+(n(n+1)2(p+q)×1∑i=1n∑j=in(1p/(wiGiγ)+q/(wjGjγ)))1γ)=1(1+(3(3+1)2(2+1)×1∑i=13∑j=i3(12/(wiGi3)+q/(wjGj3)))13)=0.3404

*Finally,* ${\mathrm{IVIFWDGHM}}_{w}^{2,1}({\tilde{\delta }}_{1},{\tilde{\delta }}_{2},{\tilde{\delta }}_{3})=\left(\left[0.2449,0.4731\right],\left[0.1734,0.3404\right]\right)$

*Then, we give some properties of the IVIFWDGHM operator, and the proof is similar to IVIFWDHM.*

**Property** **9.** *(Monotonicity) Let* ${\tilde{\delta }}_{i}=\left(\left[b_{i},d_{i}\right],\left[e_{i},g_{i}\right]\right)(i=1,2,\ldots ,n)$ *and* ${\tilde{\theta }}_{i}=\left(\left[r_{i},h_{i}\right],\left[m_{i},f_{i}\right]\right)$ (i=1,2,…,n) *be two sets of IVIFNs. If* $b_{i}\leq r_{i},d_{i}\leq h_{i}\;and\;e_{i}\geq m_{i},g_{i}\geq f_{i}$ *hold for all* i *, then,*

(84) $${\mathrm{IVIFWDGHM}}^{p,q}\left({\tilde{\delta }}_{1},{\tilde{\delta }}_{2},\cdots ,{\tilde{\delta }}_{n}\right)\leq {\mathrm{IVIFWDGHM}}^{p,q}\left({\tilde{\theta }}_{1},{\tilde{\theta }}_{2},\cdots ,{\tilde{\theta }}_{n}\right)$$

**Property** **10.** *(Boundedness) Let* ${\tilde{\delta }}_{i}=\left(\left[b_{i},d_{i}\right],\left[e_{i},g_{i}\right]\right)(i=1,2,\ldots ,n)$ *be a set of IVIFNs. If*

${\tilde{\delta }}_{\max}=\left(\left(\left[\max_{i}\left(b_{i}\right),\max_{i}\left(d_{i}\right)\right],\left[\min_{i}\left(e_{i}\right),\min_{i}\left(g_{i}\right)\right]\right)\right)$ *,*

${\tilde{\delta }}_{\min}=\left(\left[\min_{i}\left(b_{i}\right),\min_{i}\left(d_{i}\right)\right],\left[\max_{i}\left(e_{i}\right),\max_{i}\left(g_{i}\right)\right]\right)$

*and,* ${\tilde{\delta }}^{+}={\mathrm{IVIFWDGHM}}^{p,q}({\tilde{\delta }}_{\max},{\tilde{\delta }}_{\max},\ldots ,{\tilde{\delta }}_{\max})$ *,* ${\tilde{\delta }}^{-}={\mathrm{IVIFWDGHM}}^{p,q}({\tilde{\delta }}_{\min},{\tilde{\delta }}_{\min},\ldots ,{\tilde{\delta }}_{\min})$ *then,*

(85) $${\tilde{\delta }}^{-}\leq {\mathrm{IVIFWDGHM}}^{p,q}({\tilde{\delta }}_{1},{\tilde{\delta }}_{2},\ldots ,{\tilde{\delta }}_{n})\leq {\tilde{\delta }}^{+}$$

## 4. Example and Comparison

### 4.1. Numerical Example

The forest ecological tourism demonstration area has a variety of functions, which is an important part of the national ecological tourism demonstration area. It is also destined to the forest ecological tourism demonstration area of ecological value. The corresponding study of forest ecological tourism demonstration area of ecological value is important for the promotion of human welfare and social sustainable development. That is to say, the assessment of the ecological value of the forest ecological tourism demonstration area is one of the hot spots and key issues of the domestic and international ecology academic circles and the society. At present, the current urbanization, rapid industrialization, environmental pollution situations are grim, and there are many people living with less realistic conditions of these three heavy squeezes. This highlights that the research of forest ecological tourism demonstration area of ecological value has become even more urgent. The problems of evaluating the ecological value of forest ecological tourism demonstration areas are classical MADM problems [66,67,68,69,70,71,72,73,74,75]. Thus, we give an example to solve the MADM for evaluating the ecological value of forest ecological tourism demonstration areas with IVIFNs. There are five possible forest ecological tourism demonstration areas $A_{i}\left(i=1,2,3,4,5\right)$, to been assessed. The experts use four evaluation attributes to assess five forest ecological tourism demonstration areas: (1) G_1_ is the tourism and leisure value; (2) G_2_ is the material production value; (3) G_3_ is the scientific research and cultural value; (4) G_4_ is the climatic regulation value. The five possible forest ecological tourism demonstration areas are to be assessed with IVIFNs (attributes weight w=(0.4,0.2,0.3,0.1)), as shown in the Table 1.

Then, we use the approach developed for selecting the best forest ecological tourism demonstration area.

**Step 1.** According to IVIFNs $r_{ij}\left(i=1,2,3,4,5,j=1,2,3,4\right)$, we fuse all the IVIFNs $r_{ij}$ by IVIFWDHM (IVIFWDGHM) operator, to calculate the IVIFNs $A_{i}\left(i=1,2,3,4,5\right)$ of the forest ecological tourism demonstration area $A_{i}$. Let p=2,q=1,γ=3, then the fused values are depicted in Table 2.

**Step 2.** By Table 2, the score results of the forest ecological tourism demonstration areas are in Table 3.

**Step 3.** By Table 3, the order of forest ecological tourism demonstration areas is listed in Table 4. The best forest ecological tourism demonstration area is A_3_.

### 4.2. Influence Analysis

**Figure 1:**
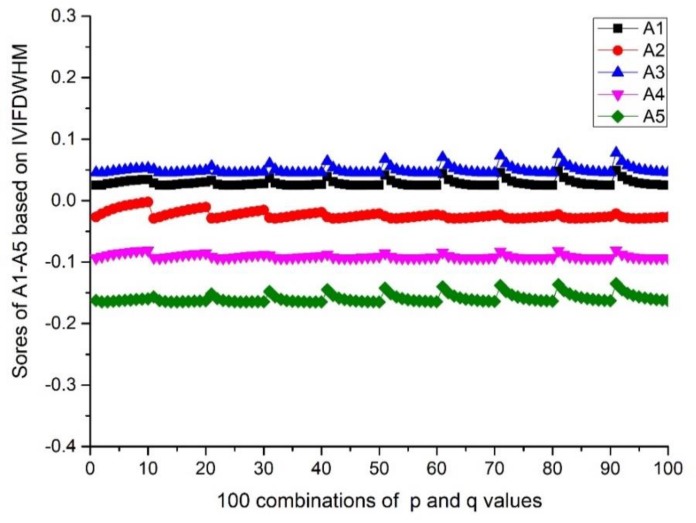
Scores of A_*i*_ (*i* = 1,2,3,4,5) based on the IVIFWDHM operator (*λ* = 3) for different integer *p* and *q* ∈ [1, 10].

**Figure 2:**
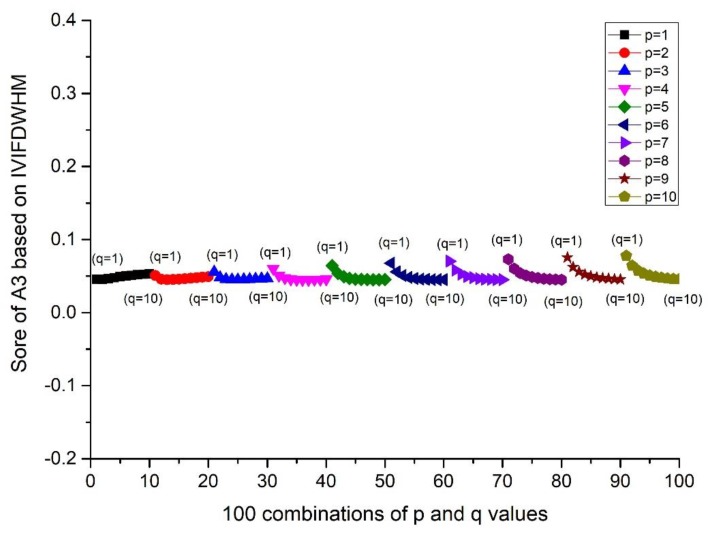
Score of A_3_ based on the IVIFWDHM operator (*λ* = 3) for different *p* and *q* ∈ [1, 10] when *p* is fixed and *q* changes from 1 to 10.

**Figure 3:**
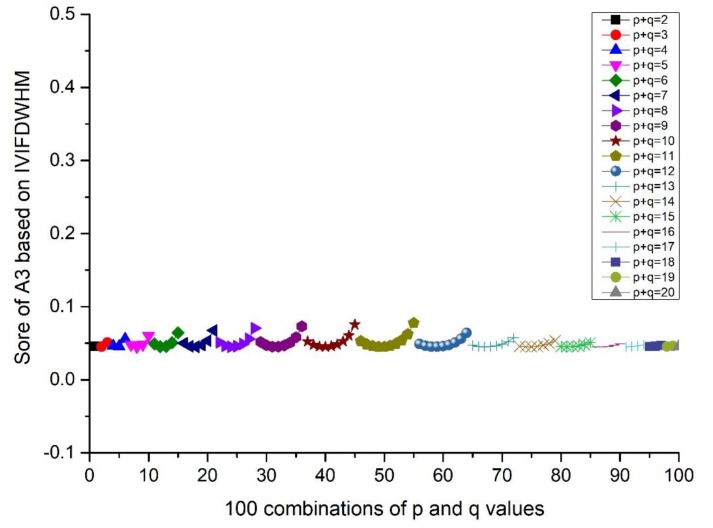
Score of A_3_ based on the IVIFWDHM operator (*λ* = 3) for different *p* and *q* ∈ [1, 10] when *p* + *q* changes from 2 to 20.

**Figure 4:**
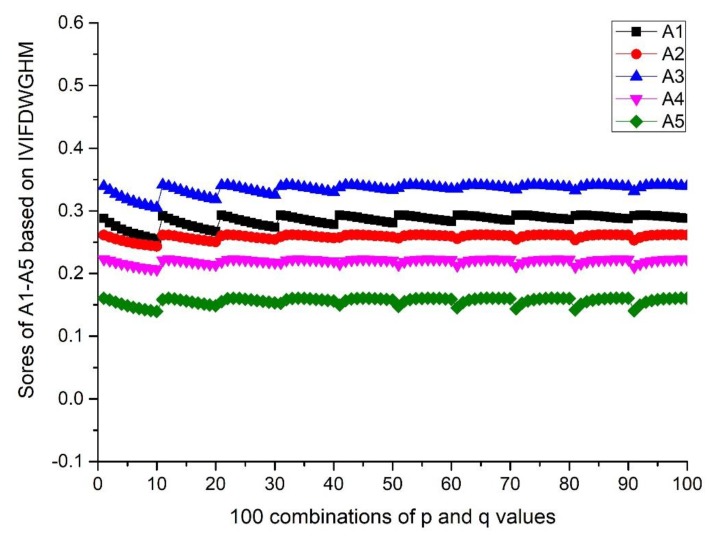
Scores of A_*i*_ (*i* = 1,2,3,4,5) based on the IVIFWDGHM operator (*λ* = 3) for different integer *p* and *q* ∈ [1, 10].

**Figure 5:**
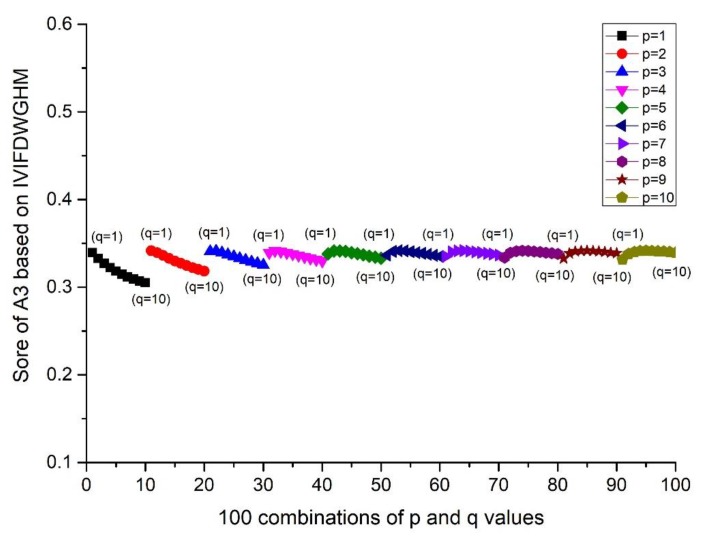
Score of A_3_ based on the IVIFWDGHM operator (*λ* = 3) for different *p* and *q* ∈ [1, 10] when *p* is fixed and *q* changes from 1 to 10.

**Figure 6:**
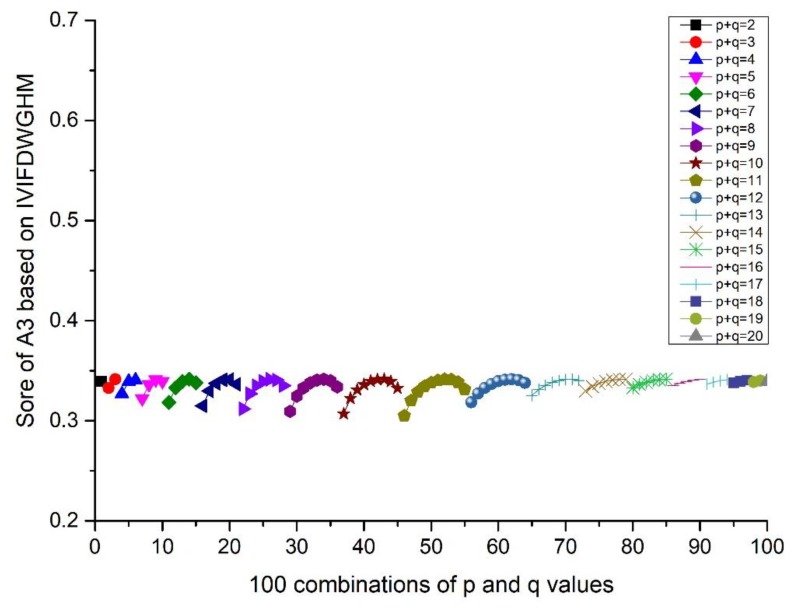
Score of A_3_ based on the IVIFWDGHM operator (*λ* = 3) for different *p* and *q* ∈ [1, 10] when *p* + *q* changes from 2 to 20.

The proposed methods have two independent parameters, p and q, which play an important role in the calculation of the results. Hence, different score values and orders may be derived when p and q change. Furthermore, the integer values of p and q in the range of 1–10 usually receive more attention in practical applications. We investigated the influences of p and q on the decision-making from the results of the IVIFWDHM operator and the IVIFWDGHM operator. Firstly, the different p and q are assigned in a certain order with $\left(p_{i},q_{j}\right)$ (i=1,2,⋯,10; j=1,2,…,10). The scores and ranking results of $A_{i}\left(i=1,2,3,4,5\right)$ are given in Figure 1 and Figure 4. Then, the influence of q (or p) on the score is investigated from the result of $A_{3}$, when the p value is fixed and q changes from 1 to 10. Details can be found in Figure 2 and Figure 5. Moreover, the influence of p+q on the score is investigated from the result of $A_{3}$, when p+q changes from 2 to 20. Details can be found in Figure 3 and Figure 6.

According to Figure 1 and Figure 4, we can conclude that different scores of alternatives can be derived according to different p and q. The differences between the maximum and minimum scores of A_1_ to A_5_ from the IVIFWDHM operator are 0.0243, 0.0265, 0.0326, 0.0132 and 0.0296, respectively, and the differences between the maximum and minimum scores of A_1_ to A_5_ from the IVIFWDHM operator are 0.0385, 0.0187, 0.0365, 0.0164 and 0.0212, respectively. It can be seen that the fluctuation range of the scores from the IVIFWDHM operator and the IVIFWDGHM operator are small. Different p and q values have little effect on the scores of the two methods for A_1_ to A_5_, so the scores of IVIFWDHM operator and IVIFWDGHM operator are stable for different p and q values. However, A_1_ to A_5_ show some variation rules for different p and q values. The following takes the scores of A_3_ from the IVIFWDHM operator and the IVIFWDGHM operator as examples to show the variation rules: (1) For the IVIFWDHM operator, Figure 2 shows that when the p value is fixed and the q value changes from 1 to 10, the fluctuation trend of the score is more complex, most of which has a decreasing trend; Figure 3 shows that when the p+q value changes from 2 to 20, the fluctuation range increases first and then decreases, and reaches its maximum when the p+q value equals 11, then the fluctuation range is from 0.0450 to 0.0776 when p+q value equals 11, which is the same as that of the scores about the 100 combinations of A_3_. But the average score of each group has little difference under a certain p+q value, the difference between the maximum average score and the minimum average score is 0.0070, while the fluctuation range is from 0.0458 to 0.0528, which is only 21.45% of the amplitude of the fluctuation range about the 100 combinations of A_3_. (2) For the IVIFWDGHM operator, Figure 5 shows that when the p value is fixed and the q value changes from 1 to 10, the fluctuation trend of the score is more complex, and the decreasing trend is dominant; Figure 6 shows that when p+q value changes from 2 to 20, the fluctuation range increases first and then decreases, and reaches its maximum when p+q value equals 11, then the fluctuation range is from 0.3050 to 0.3414, which is the same as that of the scores about the 100 combinations of A_3_. But the average score of each group has little difference under a certain p+q value, the difference between the maximum average score and the minimum average score is 0.0075, while the fluctuation range is from 0.3050 to 0.3414, which is only 20.46% of the amplitude of the fluctuation range about the 100 combinations of A_3_.

In this section, the influence of p and q on the scores are investigated for the IVIFWDHM operator and the IVIFWDGHM operator. Although results illustrate the regularity of the proposed method for the different p and q, different p and q values have little effect on the score values for the two methods_._ So the scores of the IVIFWDHM operator and the IVIFWDGHM operator are stable for different p and q values. When the scores of the subjects are similar, it is likely that the ranking of evaluation will change, but when there is a certain gap in the scores of the subjects, the ranking of evaluation will not change. Thus, the proposed methods are sufficient to solve practical MADM. Furthermore, the proposed methods show high robustness for information fusion in MADM.

### 4.3. Comparative Analysis

We compare the IVIFWDHM and IVIFWDGHM operators with the IVIFWA operator [64], the IVIFWG operator [4], the gray relational analysis method [47] and correlation coefficient [76]. The results are given in Table 5.

From the above analysis, we get the same best forest ecological tourism demonstration areas, while the four methods’ orders are slightly different. However, the existing methods with IVIFNs don’t consider the interrelationship among the arguments. Our proposed IVIFWDHM and IVIFWDGHM operators consider the interrelationship among aggregated arguments.

Xu and Chen [77] defined some Bonferroni mean for aggregating the IVIFNs. However, these Bonferroni mean for aggregating the IVIFNs only consider the relationship information between two arguments, and do not consider the relationship information among more than two arguments.

## 5. Conclusions

Traditional mass tourism attaches much importance to economic profits, while it is intended to meet the aesthetic needs of people. However, behind the high-speed development of tourism, there are difficulties in solving the relationship between man and the nature with the problems aroused in the ecological environment and resources in tourist spots. Eco-tourism is the result of advocating a harmonious coexistence between human beings and the nature, which also indicates both a new concept of tourism, and the ecological conceptions reflected in recreation behaviors of tourists. It advocates such ideas as the harmonious coexistence of man and the nature, and enjoying the nature without destroying the environment, which essentially derive from a concept of humans going back to the nature. Superficially, it comes from people’s attention to “the exterior” of traditional mass tourism, while philosophically, it suggests people’s awakening to environmental ethics. Tourist theories are becoming more mature with a change of paradigm, in which tourism is developing from the activities of a privileged minority, to a popular mass behavior, observed at present. Essentially, we perceive eco-tourism to be a kind of ecological culture based on the recognition of man’s relationship with the nature, and, that we have entered a new tourism paradigm under the guidance of eco-ethics. Furthermore, it reflects on the ideas of the traditional man-oriented mass tourism and corrects people’s misunderstanding about tourist resources and the ecological environment. In this paper, we investigated MADM with IVIFNs. Then, we utilized HM and Dombi operations to design some HM operators with IVIFNs: IVIFDHM operator, IVIFWDHM operator, IVIFDGHM operator and IVIFWDGHM operator. The main characteristic of these proposed operators were studied. Then, we employed the IVIFWDHM and IVIFWDGHM operators to propose two models for MADM problems with IVIFNs. Finally, a real experimental case for evaluating the ecological value of forest ecological tourism demonstration area was used to show the developed approach. In the subsequent studies, the extension and application of IVIFNs need to be studied in many other uncertain environments [78,79,80,81,82,83,84] and other applications [85,86,87,88,89,90].

## Figures and Tables

**Table 1:** IVIFN decision matrix.

	G_1_	G_2_	G_3_	G_4_
A_1_	([0.4,0.6], [0.2,0.3])	([0.3,0.5], [0.1,0.3])	([0.3,0.5], [0.1,0.2])	([0.1,0.3], [0.3,0.4])
A_2_	([0.2,0.5], [0.1,0.4])	([0.3,0.6], [0.2,0.4])	([0.4,0.6], [0.1,0.3])	([0.1,0.4], [0.3,0.5])
A_3_	([0.5,0.7], [0.2,0.3])	([0.3,0.6], [0.2,0.3])	([0.2,0.4], [0.3,0.4])	([0.4,0.5], [0.1,0.2])
A_4_	([0.4,0.4], [0.2,0.4])	([0.3,0.4], [0.2,0.3])	([0.2,0.4], [0.4,0.3])	([0.2,0.3], [0.1,0.2])
A_5_	([0.2,0.6], [0.2,0.4])	([0.2,0.4], [0.4,0.6])	([0.1,0.5], [0.3,0.4])	([0.3,0.6], [0.2,0.3])

**Table 2:** The fused values of the forest ecological tourism demonstration areas by IVIFWDHM (IVIFWDGHM) operator.

	IVIFWDHM	IVIFWDGHM
A_1_	([0.2296,0.4071], [0.2002,0.3791])	([0.3184,0.5926], [0.1191,0.2079])
A_2_	([0.1981,0.4259], [0.1852,0.4969])	([0.2881,0.6370], [0.1110,0.2911])
A_3_	([0.2834,0.4877], [0.2672,0.4024])	([0.4112,0.6420], [0.1474,0.2229])
A_4_	([0.2165,0.2809], [0.2730,0.4103])	([0.3643,0.4959], [0.1854,0.2324])
A_5_	([0.1325,0.4239], [0.3474,0.5223])	([0.2354,0.6262], [0.2009,0.3437])

IVIFWDHM: interval-valued intuitionistic fuzzy weighted Dombi Heronian mean; IVIFWDGHM: interval-valued intuitionistic weighted Dombi geometric Heronian mean.

**Table 3:** The score results of forest ecological tourism demonstration areas.

	IVIFWDHM	IVIFWDGHM
A_1_	0.0287	0.2920
A_2_	−0.0290	0.2615
A_3_	0.0508	0.3415
A_4_	−0.0929	0.2212
A_5_	−0.1566	0.1585

**Table 4:** Order of the forest ecological tourism demonstration areas.

	Order
IVIFWDHM	A_3_ > A_1_ > A_2_ > A_4_ > A_5_
IVIFWDGHM	A_3_ > A_1_ > A_2_ > A_4_ > A_5_

**Table 5:** Order of the tourism scenic spots.

Methods	Order
IVIFWA operator [64]	A_3_ > A_1_ > A_4_ > A_2_ > A_5_
IVIFWG operator [4]	A_3_ > A_1_ > A_2_ > A_4_ > A_5_
Gray Relational Analysis Method [47]	A_3_ > A_5_ > A_1_ > A_2_ > A_4_
Correlation Coefficient [76]	A_3_ > A_1_ > A_2_ > A_4_ > A_5_
